# Population structure and ongoing microevolution of the emerging multidrug-resistant *Salmonella* Typhimurium ST213

**DOI:** 10.1038/s44259-024-00027-6

**Published:** 2024-04-08

**Authors:** Isela Serrano-Fujarte, Edmundo Calva, Jimena García-Domínguez, Stephanie Ortiz-Jiménez, José L. Puente

**Affiliations:** Departamento de Microbiología Molecular, Instituto de Biotecnología, Universidad Nacional Autónoma de México, Cuernavaca, MOR Mexico

**Keywords:** Bacterial genomics, Pathogens, Bacterial pathogenesis

## Abstract

*Salmonella enterica* serovar Typhimurium ST213 is an emergent multidrug-resistant sequence type associated with the food chain, and gastrointestinal and invasive infections in North America. Here, we applied genomic and phenotypic analyses to illustrate the diversity and evolution of sequence type ST213. The population structure and evolutionary history of ST213 strains, particularly the North American isolates (NA-ST213) distinguish them from other *S*. Typhimurium sequence types, including European ST213 strains. NA-ST213 isolates were distributed in four co-circulating lineages with distinct multidrug resistance profiles and unique phage and CRISPR spacers patterns that could have shaped their local microevolution. Compared to the SL1344 reference strain, NA-ST213 demonstrated reduced adherence and internalization in cultured eukaryotic cell lines but exhibited more efficient replication and intracellular survival. This study underscores the relevance of studying an emergent *S*. Typhimurium sequence type and the events leading to its diversification beyond the well-characterized reference strains and worldwide predominant sequence types. However, it must also serve as a cautionary tale of the potential health risk the NA-ST213 may represent; particularly when there is a close relationship with pandemic sequence types such as the monophasic ST34.

## Introduction

*Salmonella enterica* serovar Typhimurium is a broad host range pathogen, primarily associated with enteric-limited infections such as gastroenteritis in humans and animals. However, *S*. Typhimurium can also cause severe invasive infections in children, older individuals, and immunocompromised individuals^1,2^.

Multilocus sequence typing (MLST) has been used to better understand the global epidemiology of this pathogen. This approach revealed that the *S*. Typhimurium isolates belong to a single clonal complex, and the most abundant sequence type (ST) is the ST19 sequence type^3^. Nonetheless, recently, there has been an increase in the reports of emerging sequence types dominating geographical areas, such as the ST34 sequence type, which was initially associated with gastrointestinal infection in Europe and is currently found to be globally distributed as a pandemic clone^4,5^. Other sequence types of interest are ST313 and ST213. The ST313 sequence type is able to cause severe bloodstream infections in immunocompromised individuals in Africa and was recently identified in the United Kingdom and Brazil^6^. The ST213 sequence type is replacing the dominant ST19 sequence type in Mexico and is linked to gastrointestinal and invasive infections^7–9^, and it has also been reported in the USA (United States of America), Canada, and the UK (United Kingdom).

*S*. Typhimurium ST213 strains isolated in North America have remarkable characteristics, such as the lack of the virulence-associated plasmid pSTV (also called pSLT), which is broadly distributed in the serovar Typhimurium; the predominant presence of plasmids belonging to the IncC family; and a multidrug-resistant (MDR) profile, including resistance to third-generation cephalosporins like ceftriaxone, the treatment of choice for acute gastroenteritis or complicated invasive infections^6,9–11^. Notably, in Mexico, MDR-*S*. Typhimurium strains have been associated with the higher frequency of invasive infections and mortality in children compared with non-MDR strains^7^. The association of this sequence type with some cases of invasive infections in immunocompetent individuals, in addition to the high levels of antimicrobial resistance that narrow treatment options^10^, represents a major health concern. The strong correlation found between human infections and contaminated beef with ST213 strains^8^, suggest that this sequence type takes advantage of a widespread transmission network allowing its prevalence throughout the food chain. Moreover, this sequence type has been identified in other food sources, such as cheese, indicating its ability to persist in different environments and tolerate diverse stresses^12^. Furthermore, recent studies have reported the isolation of ST213 from surface waters commonly utilized in agriculture^13^, highlighting its potential for transmission through multiple routes.

Our investigation aims to phylogenetically and phenotypically characterize ST213 strains isolated in North America and identify specific genetic elements currently driving their expansion. We hypothesize that the lack of the pSLT virulence plasmid and the acquisition of distinct genetic elements, including IncC plasmids carrying antimicrobial determinants, have played a pivotal role in the ongoing expansion and prevalence of ST213 strains in North America. Gaining insights into the molecular determinants and phenotypic characteristics contributing to the prevalence and spread of ST213 sequence type in the region, may draw attention to the necessity of implementing epidemiological and antimicrobial surveillance strategies. Coupled with the study of *S*. Typhimurium ST213 strains’ survival and virulence features, this approach aims to explore this emergent sequence type in a broader context, beyond the reference strains.

## Results

### The population structure of the *S*. Typhimurium ST213 shows intra-sequence type evolution

To establish and understand the population structure of the ST213 sequence type, 275 *S*. Typhimurium ST213 genomes publicly available in the EnteroBase database^3,14^, were used to build a whole-genome single-nucleotide polymorphism (SNP) phylogenetic analysis, and cluster designation was performed using FastBAPs^15^. This sample includes 43 *S*. Typhimurium ST213 collected in México between 2002 and 2005 during an integrated food chain surveillance program (Zaidi collection)^8^, that were sequenced in the context of the UoWUCC 10 K genomes project^8,14,16^. Five representative strains from this collection were fully sequenced and annotated in previous studies^17–19^. And the sample comprised 232 draft genomes of ST213 strains, collected between 1957 and 2022 from another 13 countries. Additionally, 15 reference strains representing the ST19, ST34 and ST313 sequence types were obtained from the EnteroBase or the NCBI refseq database and included in the analysis (Supplementary Table 1).

**Fig. 1:**
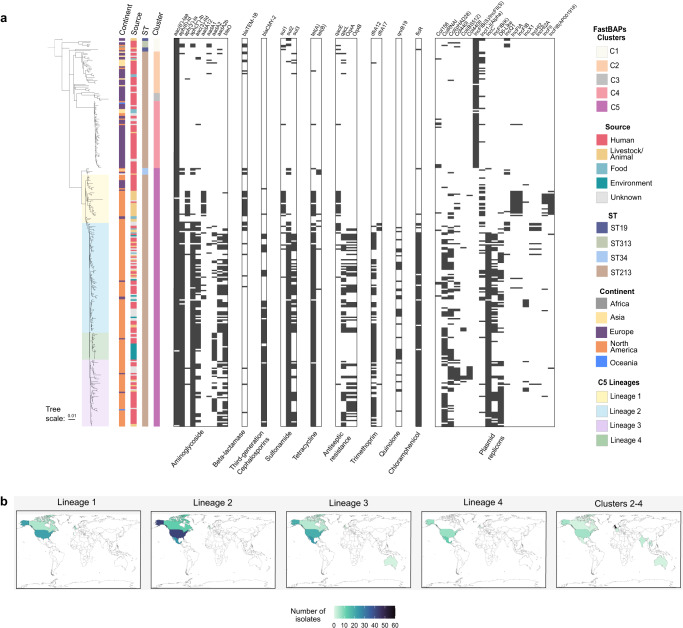
Population structure and accessory genomic repertory of the ST213 sequence type. **a** Maximum-likelihood phylogeny showing the clusters, lineages, MDR patterns, and plasmid repertory of the ST213 sequence type (*n* = 275), including five ST19 reference strains, five ST313, and five ST34 isolates. **b** Visualization of the lineages and clusters distribution of ST213 strains by country.

This analysis revealed five clusters at the FastBaps level 1, grouping in relation to their geographical distribution. The first cluster (C1) comprised the ST19 and ST313 representative strains, while the second to fourth clusters (C2, C3 and C4) included ST213 isolates mostly from Asia and northwestern Europe. The fifth cluster (C5) contained almost all northern American isolates (96.6%), and only 16% of isolates from other continents. As a result, it emerged as the major cluster containing 68% of the total strains; surprisingly this cluster also included the ST34 representative strains incorporated in the analysis (Fig. 1a, b). For discussion reasons, the C2, C3, and C4 clusters will be referred to as EU-ST213 (northwestern Europe and Asian isolates), and the ST213 in the cluster C5 will be referred to as NA-ST213 (Canada, USA, and Mexico isolates).

Despite of the NA-ST213 distribution cluster across three countries in North America, it is noteworthy that all strains lacked the virulence plasmid and most showed extensive drug resistance carried by an IncC-like plasmid. Moreover, this cluster includes isolates associated with cases of invasive disease^7,8,17,18^. Therefore, a more comprehensive characterization of this cluster was performed, using information from level 2 of the FastBaps clustering analysis, which led to the identification of four lineages. Lineage 2 was the most represented with 43.6% (*n* = 82) of the NA-ST213 strains, followed by lineage 3 with 26.6% (*n* = 50): the isolates that formed these lineages were predominantly derived from human sources. Lineage 1 (19.1%, *n* = 36) included human isolates as the main source and has been disseminated in Canada and USA with a few isolates from UK, while lineage 4 (10.6%, *n* = 20) was primarily collected from water/rivers in the USA and Mexico (Fig. 1b).

### Accessory genome acquisition through horizontal transfer events has geographically diversified the ST213 sequence type

Following their initial identification, special attention was focused on the NA-ST213 isolates due to the multidrug-resistant profiles they exhibited^8,9,11^. To establish a correlation between the population structure and the temporal and geographic evolution of antimicrobial resistance within this sequence type, as well as its plasmid content, the genetic determinants potentially conferring the resistance were searched.

A total of 58 associated resistance genes were identified (Supplementary Data), confirming the wide antimicrobial resistance (AMR) patterns and their association with the geographical distribution of the ST213 isolates. This is consistent with a previous report^20^ also showing that the EU-ST213 strains (*n* = 87) carried fewer AMR determinants compared to the NA-ST213 strains (*n* = 188) (Fig. 1a and Supplementary Fig. 1A).

None of the EU-ST213 strains were MDR and did not exhibit increased antimicrobial profiles. Among the NA-ST213 strains, those belonging to the L1 lineage were the most sensitive (*n* = 36). Fifteen of them carried only one chromosomal gen (*aac(6’)-Iaa*), which is a ubiquitous and generally non-functional *Salmonella* gene encoding a 6′-N-acetyltransferase type I. Except for rare cases, this gene does not confer resistance to aminoglycosides^21^. In contrast, 18 of them carried genes conferring resistance to beta-lactams, sulfonamides, and tetracyclines (Fig. 1a).

Remarkably 43.6% of the NA-ST213 (82/188) displayed an MDR profile, distributed among the L2, L3 and L4 lineages. These isolates carried the plasmid-borne *blaCMY-2* gene, which confers resistance to third-generation cephalosporins, as previously shown^7,9^. Out of these isolates, 52 were also resistant to quinolones. The strains primarily associated with MDR-*blaCMY-2*-quinolone resistance, originating from México (25/52) and the USA (*n* = 22/52), were predominantly human isolates (35/52) (Fig. 1a and Supplementary Fig. 2A–C).

Coupling these results with the phylogenetic analysis, a gradual increase and maintenance in the MDR profile was observed across the NA-ST213 lineages (Fig. 1a). Furthermore, the NA-ST213 sequence type exhibited a higher number of plasmid replicons than the EU-ST213 sequence type, highlighting the potential influence of plasmid content on the multi-drug resistance phenotype. In this regard, except for L1 and a few L2 strains, L2, L3 and L4 NA-ST213 strains contained IncC plasmids (*n* = 143/188) being the main carriers of the *blaCMY-2* gene, and these plasmids are not present in any of the EU-ST213 strains (Fig. 1a and Supplementary Fig. 1B).

**Fig. 2:**
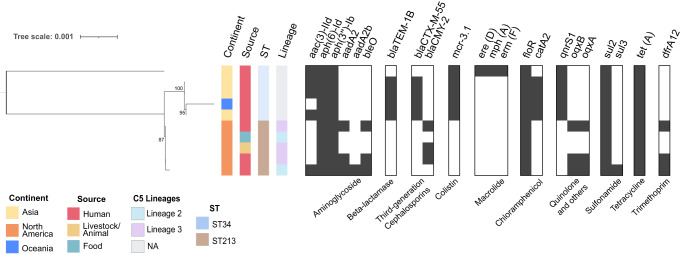
Plasmid phylogeny of IncC from ST213 and ST34 *S*. Typhimurium sequence types. SNP core genome phylogeny of assembled IncC plasmids isolated from five ST34 (NCBI accession numbers: OQ658822, OQ658820, MN647788, OU015324, NZ_CP091867) and five ST213 (NCBI accession numbers: NZ_CP035548, NZ_CP032496, NZ_CP032491, NZ_CP011429, NZ_012682). The tree was midpoint rooted and coupled with the AMR genes identified (black rectangle indicates presence and white indicates absence).

IncC plasmids exhibit a broad host range and have been identified in other *S*. Typhimurium STs, including ST34 in Asia^22,23^. A comparison between plasmids from ST34 and ST213 revealed a sharing of 92 core genes, with substantial variation in accessory genes. Analyzing a core SNP phylogeny of five NA-ST213 and five Asian IncC plasmids revealed a clustering of NA-ST213 IncC plasmids, distinct from their Asian counterparts, and were almost indistinguishable from each other. Notably, the ST34 IncC plasmids harbor distinct AMR determinants compared to NA-ST213 IncC plasmids, with only the latter containing the *blaCMY-2* gene. It is worth mentioning that IncC plasmids isolated from food and livestock lack quinolone and trimethoprim resistance genes, in contrast to those from human isolates (Fig. 2).

Interestingly, none of the NA-ST213 isolates carried the IncFIB(S) and IncFII(S) replicons from the virulence-associated plasmid pSTV, as previously shown for some isolates from Mexico and ST34 strains^9–11,22^. In contrast, EU-ST213 strains (*n* = 90/93) did contain these replicons (Fig. 1a and Supplementary Fig. 1B). This plasmid is well known for harboring virulence genes, and it is noteworthy that most strains employed in the in-depth study of *S*. Typhimurium pathogenesis carry it.

The pSTV plasmid has been broadly studied and is relevant because carries specific virulence genes involved in bacterial adhesion (*pefABCD*), invasion (*rck, spvAC*) and intracellular survival (*mig-5, spvD*)^24–28^. The lack of the pSTV do not necessarily implies the absence of the virulence genes associated to it, some genes could be detected in other plasmids or in the chromosome^29,30^; however, using the virulence finder database (VFDB)^31^, we showed that all the NA-ST213 isolates lacking the pSTV plasmid did not carry the virulence genes associated with it (Supplementary Fig. 3).

The NA-ST213 strains lack other virulence determinants particularly the Gifsy-1 and in a few cases, Gifsy-2 prophages that play a role in *Salmonella* virulence: *Gifsy*-2 is central to the intracellular survival, whereas *Gifsy*-1 contributes to a lesser extent in the colonization stage^32^. Interestingly, all NA-ST213 isolates lack the *Gifsy*-1 prophage, but not the ST34 isolates. The NA-ST213 strains, except lineage 1 isolates, displayed a diverse assortment of a subset of both complete and incomplete versions of several prophages, including SE1, UAB_Phi20, P88, 186, and P1, which were notably absent in the EU-ST213, ST19, ST34, and ST313 isolates included in this study (Supplementary Fig. 4). No genes involved in virulence were identified in these prophages.

**Fig. 3:**
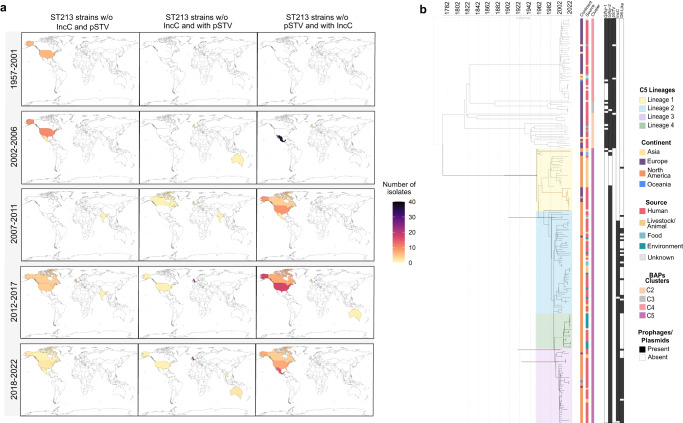
Spatiotemporal analysis of ST213 sequence type. **a** Visualization of the spatiotemporal distribution analysis of the pSTV and IncC plasmids harbored by ST213 isolates. **b** RelTime analysis showing a timed phylogeny of 218 ST213 isolates, coupled with key accessory genome content. Confidence intervals (95% CI) of relevant nodes are presented in red (To consult all the nodes with 95% CI refer to Supplementary Data).

The presence of the IncC plasmids in the NAST213 isolates led us to investigate their acquisition. A spatiotemporal view of the isolates with these plasmids revealed that they first appeared between 2002 and 2006 in Mexico and continued expanding across northern America, showing limited to no distribution to other continents throughout the years (Fig. 3a). In accordance with our observations, the IncC plasmids have been mainly associated as carriers of antimicrobial resistance genes^11^, and their implications in bacterial virulence and physiology are yet to be explored.

### Historic reconstruction of the *S*. Typhimurium ST213 sequence type reveals a potential trigger for NA-ST213 lineage 2 expansion

To investigate the spread and diversification of the ST213 sequence type through its evolutionary history a timetree, using the RelTime with dated tips method, was inferred in MEGA^33^. The most-recent common ancestor (MRCA) of all ST213 may have emerged approximately in 1771 (95% confidence interval (CI) 1608–1858 (Fig. 3b and Supplementary Data). The EU-ST213 diverged around 1813 (95% CI 1701–1884) into three clusters; the C3 cluster diverged around 1819 (95% CI 1701–1909), followed by C2 around 1931 (95% CI 1852–1969) and more recently C4 diverging around 1999 (95% CI 1964–2000).

The NA-ST213 lineages started to diverge around 1954 (95% CI 1893–1956). Lineage 1 comprised the oldest strains in the dataset (1957, 1965, and 1988) and was mainly isolated in the USA from livestock between 2000 and 2006, the isolates from Canada (*n* = 5) and United Kingdom (UK, *n* = 7) were primarily isolated from humans between 2014 and 2021. Remarkably these latest isolates had the *Gifsy-2* prophage absent in the early isolates from USA, and they seem to fit into a discrete sub-lineage inside lineage 1, showing how the accessory genome acquisition and lost by this sequence type is highly dynamic (Fig. 3b). Lineages 2 and 3 diverged nearly in 1957 (95% CI 1893–1979) and 1989 (95% CI 1919–2000), respectively, their oldest isolate being from 2002. We could not find lineage 3 isolates after 2018, while lineage 2 remained the most frequent isolate in the last 5 years compared to all lineages. These two lineages are the most abundant in the dataset and the isolates from the Zaidi collection were in these categories (Lineage 2 *n* = 19, Lineage 3 *n* = 24). Lineage 4 emerged most recently, diverging in ~1991 (95% CI 1924–2000). The first isolate of this emergent lineage was collected in 2012 and isolations began to increase between 2018 and 2021, suggesting a potential replacement of lineage 3 (Fig. 3b and Supplementary Data).

As described above, except for all isolates from lineage 1, nine from lineage 2, and only one from lineages 3 and 4, most NA-ST213 strains carry IncC plasmids (Fig. 1a). Associating these data with the timetree, it is plausible to consider that the acquisition of these plasmids could have favored the expansion of lineages 2, 3, and 4. The presence of these plasmids may have conferred the ability to survive exposures to antibiotics in various settings, including hospitals, the agricultural use of antibiotics in livestock farming, and even the environment. This broad exposure to antibiotics could have played a crucial role in increasing the transmission of these lineages, further contributing to their expansion (Fig. 3b). Unfortunately, due to the absence of complete geospatial data and full clinical information, a precise transmission dynamic of this sequence type cannot be determined.

### NA-ST213 isolates show low internalization to eukaryotic cells but high intracellular replication rates

To explore whether representative strains of lineages 2 and 3, which are the most prevalent in the NA-ST213 strain collection and include the strains isolated from invasive cases, also exhibit distinctive phenotypic traits compared to reference strains of the founder sequence type ST19, we tested in vitro phenotypes associated with survival in the environment and host interaction.

To explore the pathogenesis of these strains, human macrophage-like cells (U-937 and THP-1), human enterocyte-like cells (C2BBe1 and HT-29), and murine fibroblasts (MEFs 3T3) were infected with four human isolates (two lineage 2 and two lineage 3, from gastrointestinal or invasive disease).

**Fig. 4:**
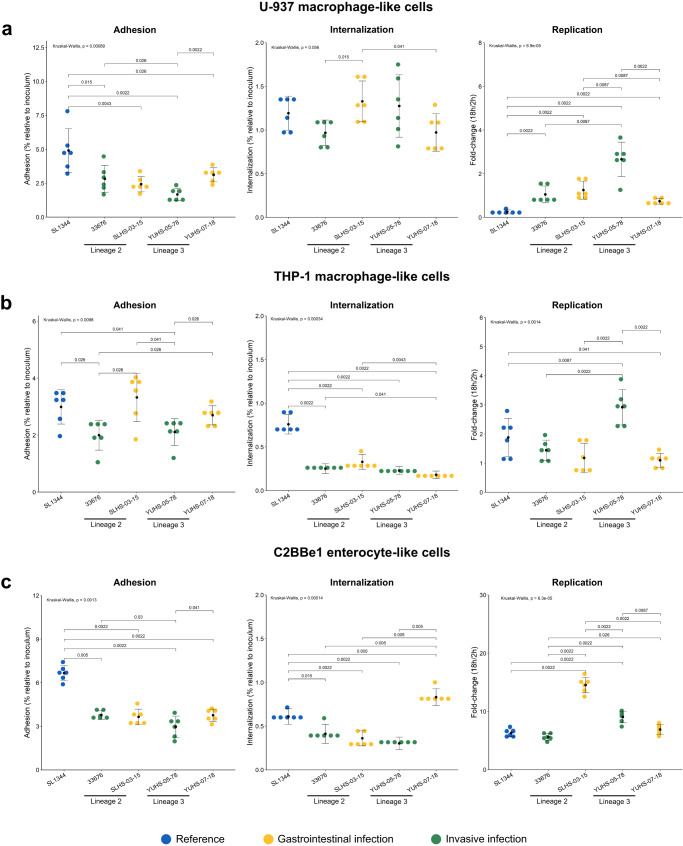

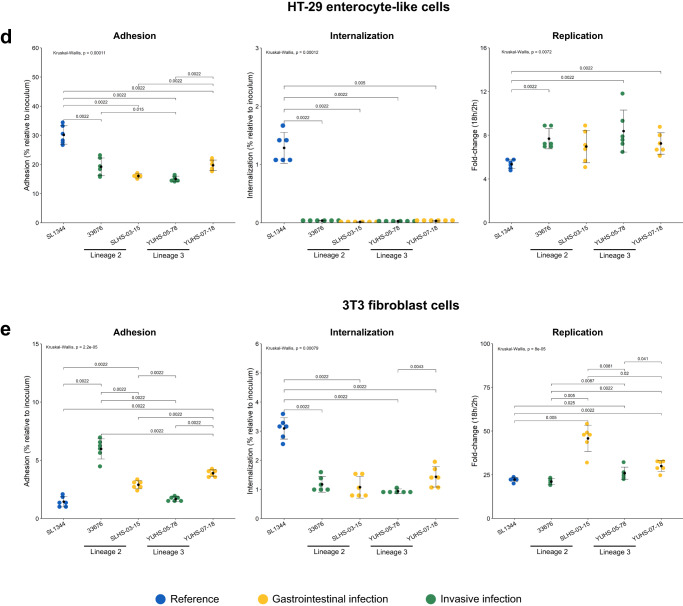
NA-ST213 adhesion, internalization, and replication in eukaryotic cells. U-937 (**a**) THP-1 (**b**) macrophage-like cells, C2BBe1 (**c**) HT-29 (**d**) enterocyte-like cells, and MEFs 3T3 fibroblasts (**e**) were infected with NA-ST213 isolates. For the adhesion assays, all the cell lines were immobilized with cytochalasin D and infected for 30 min. The internalization and replication were measured employing aminoglycoside protection assays, the cells were incubated for 2 h (internalization) or 18 h (replication) with the bacterial culture, then lysed and counted (Colony-forming units/ml). Each dot represents an independent biological replicate, and the error bars indicate the standard deviation of *n* = 6 replicates. Blue ST19 reference strain, green isolates from systemic diseases and yellow isolates from gastrointestinal disease. Groups were compared using Wilcoxon test and the *p* values are indicated in each graphic.

All the isolates exhibited a diminished ability to adhere to human cell lines compared to the SL344 reference ST19 strain (Fig. 4a–d), with the exception of two isolates from gastrointestinal infection that showed increased adhesion to THP-1 cells (Fig. 4b). In contrast to human cell lines, all the isolates showed increased or similar adhesion to murine fibroblasts compared to the reference strain (Fig. 4e). NA-ST213 isolates demonstrated reduced internalization in THP-1 macrophage-like and epithelial cells as compared to ST19 (Fig. 4b–e). To verify that these phenotypes were consistent with NA-ST213 strains, the ability to internalize by six more isolates was tested in selected cell lines, confirming that is a generalized characteristic of this sequence type (Supplementary Fig. 5A, B). In the case of U-937 macrophage-like cells, all the isolates were internalized at the same level as the reference strain (Fig. 4a), this could be due to the reported enhanced phagocytosis of this cell line^34,35^, making negligible the pathogen-induced macropinocytosis. It should be noted that these isolates were not impaired in their ability to swim and to secrete the effectors required to enter eukaryotic cells (Supplementary Figs. 6 and 7).

Remarkably, the replication rates of the NA-ST213 isolates were the same or higher than those of the reference strain (Fig. 4a–e). In the cells where the internalization was less compromised such as C2BBe1 and THP-1, the NA-ST213 isolates achieved the same bacterial load as the reference strain. However, in fibroblast and HT29 cells, the NA-ST213 isolates reached only half of the bacterial load observed with the ST19 isolate. Considering that the internalization in HT-29 cells was seven times lower than for the reference strain, the replication rate appeared to successfully compensate for this impairment (Supplementary Fig. 8).

The higher replication rates did not affect the cell viability, nor the reduced internalization showed less cytotoxicity at early stages of infection compared to the reference strain (Supplementary Fig. 9). Although subtle variations in these phenotypes were observed between the NA-ST213 strains no distinctive features were detected between lineages or infection type. In general, the isolates showed reduced adhesion and internalization capacity in human cells but seemed to atone for this deficiency with higher replication rates without affecting cell viability. This observation is crucial for understanding the pathogenesis of this emergent sequence type that is spreading across North America. It underscores the need to implement measures for its monitoring and control.

### Biofilm formation ability and Congo red binding of NA-ST213 human isolates

During the analysis of the IncC plasmids, we found that four out of the five assembled NA-ST213 plasmids contain two putative proteins related to cyclic di-GMP (c-di-GMP) production (diguanylate cyclase) and degradation (phosphodiesterase). High concentrations of c-di-GMP in the cell stimulate the expression of the master regulator CsgD, thus promoting biofilm formation^36^.

To investigate whether these proteins could directly impact the biofilm phenotype, the red, dry, and rough (RDAR) morphotype was performed on ten representative strains from lineages 2 and 3 in the Zaidi collection. These strains included one without the IncC plasmid (SLHS-03-15), three carrying the IncC plasmid with only the putative diguanylate cyclase gene (YUHS-02-75 and YUHS-05-26 a/b), and seven with the IncC plasmid and both genes. Three ST19 reference strains (ST4/74, SL1344, and 14028) were also included.

Nine of the NA-ST213 strains and two of the three ST19 reference strains exhibited the RDAR morphotype. To explore in detail the RDAR macrocolonies, a semi-quantitative approach allowed us to identify subtle structural differences among the isolates, leading to their classification into four groups based on their architecture (Supplementary Fig. 10A).

**Fig. 5:**
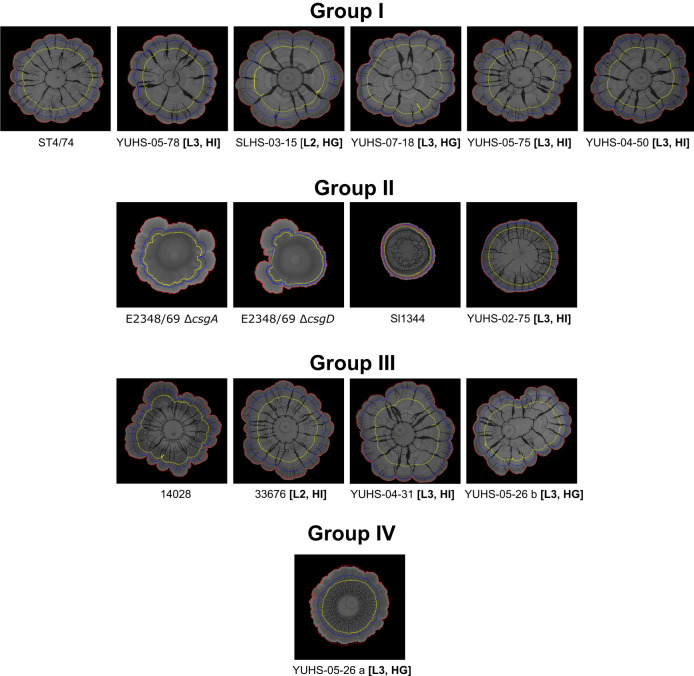
RDAR morphotype. RDAR macrocolonies were grouped according to the clustering results (Supplementary Fig. 10), the red contour indicates the size of the macrocolony at 12 days, blue at 7 days and yellow at 5 days. The letters in bold indicate the lineages (L2: lineage 2 and L3: lineage 3) and the infection type (HI human invasive and HG human gastrointestinal).

Macrocolonies with larger areas were categorized in groups I and III. However, group III macrocolonies showed more wrinkles and rings compared to those in group I. In contrast, group II comprised smaller colonies, two of which exhibited wrinkles and rings, while group IV included only one isolate with an average area and abundant wrinkles (Fig. 5).

Alternatively, we also confirmed that this ability was extensive to biofilm formation in liquid cultures on polystyrene surfaces. In general, the grouping was similar to that of the RDAR morphotype (Supplementary Fig. 11), proving the capacity of nine NA-ST213 strains to form biofilms at variable levels. However, we were unable to identify signature RDAR characteristics that allowed us to classify the NA-ST213 isolates based on lineage, isolation source, or IncC plasmid presence, implying that the phosphodiesterase and diguanylate cyclase in this plasmid did not play an apparent role in biofilm formation under the tested conditions.

## Discussion

Non-typhoidal *Salmonella* plays an important role as the causative agent of invasive and gastrointestinal infections globally^37^. The *S*. Typhimurium NA-ST213 sequence type is mainly distributed across North America and has been linked to gastrointestinal and sporadic invasive infections^8,10^, but continues to be isolated from animals, food, and water^8,13,38,39^. Here we revealed the population structure of the ST213 sequence type, showing the co-circulation of multiple lineages in North America. By linking the accessory genome content with the phylogenetic analysis, we identified a clear geographical distribution between north America (NA-ST213) and Europe (EU-ST213) isolates, as reported elsewhere^20^ (Fig. 1). This observation has led us to speculate that these strains share the ST213 sequence type by chance, which strengthens the notion that the MLST method lacks the required depth to analyze the microevolutionary progression of this sequence type, as well as possibly other emerging sequence types.

We did not find shared accessory genome signatures between EU-ST213 and NA-ST213 isolates. In this matter, EU-ST213 strains were closest to the ST19 reference strains, while NA-ST213 strains share specific traits with the ST34 sequence type such as the presence of CRISPR spacers HadB20, STM31 and STMB34, and the absence of spacers STMB20 to STMB31 (Supplementary Fig. 12), two CRISPR-associated pseudogenes (*casA* and *cas3’*, Supplementary Fig. 13) and the lack of the virulence plasmid pSTV. In addition, the NA-ST213 isolates showed distinctive features such as the lack of the *Gifsy-1* prophage and the presence of IncC plasmids (Fig. 1 and Supplementary Fig. 4).

Remarkably, the acquisition of IncC plasmids shapes part of the historic reconstruction of NA-ST213 strains, a spatiotemporal analysis with the currently available data indicates that NA-ST213 lineage 2 and 3 acquired the IncC plasmids in Mexico. This could improve their transmission due to the plasmid-encoded MDR genes and therefore led to a greater expansion of these lineages compared with lineage 1 (Fig. 3a, b). However, strains belonging to lineage 1, which do not contain plasmids of the IncC family nor the pSTV, have recently emerged containing the *Gifsy-2* prophage, which is relevant for the pathogen virulence, but is absent in the oldest isolates of this lineage, whether this could lead to intensify their expansion remains to be seen (Supplementary Fig. 4).

The association between AMR acquisition and increased transmission has been previously reported in other *Salmonellae*, leading to the clonal replacement of susceptible lineages^40,41^. It is also known that the IncC plasmids of the ST213 sequence type have a very low conjugation frequency and this has been associated with clonal dissemination^11^, these results are in agreement with our historic reconstruction data, and represent a reminder of the relevance of the pathogen study in the context of their population structures (Fig. 3).

Considering the IncC plasmid not only as a carrier of AMR determinants but also as a bearer of genes capable of modifying bacterial physiology, we evaluated the formation of the RDAR morphotype. Although is not a virulence adaptation, it has been hypothesized to be relevant for transmission and persistence^42,43^. Most of the NA-ST213 isolates tested showed all the characteristics of the RDAR morphotype and a high concentration of CR dye binding. We observed slight differences between the macrocolonies of NA-ST213 isolates, not only in architecture but also in the patterns of CR binding (Fig. 5 and Supplementary Fig. 10a–c). These differences could be attributed to the varying behavior of the mechanisms controlling curly production and CR binding among the analyzed strains, as reported for *E. coli* O157:H7^44^.

Further studies are needed to understand the factor driving RDAR morphotype diversity in this sequence type and to determine whether there is an association with the genomic characteristics described in this study.

Furthermore, these strains contain other plasmids that have been considered of interest, for example, the NA-ST213 strains isolated in Mexico have been associated with the D6-like prophage plasmid^19^, and our analysis revealed that this plasmid is also harbored by isolates from the USA and Canada although to a lesser extent. Nonetheless, even if this plasmid may play an important role in the context of the ST213 sequence type, it is not widely distributed, as only 25% of the isolates analyzed here harbored the plasmid and was mainly distributed between NA-ST213 lineages 2 (39%) and 3 (76%) and lost in the genetically younger lineage 4 (Fig. 3b). Also, in this study, we assessed different phenotypic characteristics of four isolates, two that harbor the D6-like plasmid (lineage 3) and two that lack it (lineage 2), and no differences were found (Figs. 4 and 5).

Part of the relevance of the NA-ST213 sequence type is their potential ability to cause invasive infections in humans lacking well-characterized virulence determinants, including the virulence plasmid (pSTV)^10^ and their virulence-related genes (Fig. 1 and Supplementary Fig. 3). Similar observations have been made for the epidemic clones of monophasic *S*. Typhimurium ST34 mainly linked to gastrointestinal infections in humans and animals^4,45,46^. Our findings suggest that the lack of virulence determinants does not reduce the NA-ST213 strains capabilities to cause disease (Fig. 4a–e). These strains showed reduced adhesion and internalization, but increased replication rates than the reference strain in eukaryotic cells, similar to the reports for invasive isolates of *S*. Typhimurium ST313 sequence type in Brazil^47^, *S*. Typhimurium ST34 isolates (high replication intra-sequence type)^48^ and the human-restricted *S*. Typhi (low adhesion and invasion)^49^. In contrast, studies with other ST213 isolates from food have shown high internalization in undifferentiated epithelial cells^39^, this could be related to the cell line used or isolation source, but it is unknown how these isolates fit into the population structure of the ST213 sequence type, therefore, further research is needed.

This result has led us to the hypothesis, that NA-ST213 isolates may use the lower invasion levels to avoid the immune system eliciting a response more similar to the delayed *S*. Typhi infection^50^ and then compensating for the bacterial load through higher replication rates. This could be supported by previous experiments conducted in murine models where ST19 reference strains outcompete NA-ST213 human invasive isolates in the first 3 days of infection^10^.

The NA-ST213 strains have similar phenotypic behaviors, nonetheless some of these isolates possess unique genome signatures, for example, two of the lineage 3 isolates in our study (YUHS-05-78 and YUHS-07-18) lack the immunogenic outer membrane protein OmpD, and this do not affect the ability of the bacteria to internalize and survive in human macrophages (Supplementary Fig. 14). The lack of this protein may have implications in vaccine development since it has been suggested as a candidate against NTS^51,52^. However, none of the other isolates studied here lacked this protein, demonstrating once more the diversity of *S*. Typhimurium and its implications in their pathogenic potential.

Our data highlight the diversity of the ST213 sequence type supporting that the depth of current typification methods, such as MLST, are no longer adequate to characterize emergent sequence types. Taking this into consideration, we performed CRISPR subtyping to obtain information about the geographical distribution and microevolution of the ST213 sequence type. We then coupled AMR determinants, virulence factors, plasmid, and prophage data with the population structure to understand the dynamic changes that this pathogen faces over its evolutionary history. Our study reveals compelling evidence of a seeming close relationship between the pandemic ST34 monophasic *Salmonella* sequence type, which shows a MDR profile and is one of the leading causes of gastrointestinal infections in Europe, Asia and Oceania^46,48,53^, and the emergent NA-ST213 sequence type, shedding light on potential health risks.

These findings emphasize the importance of implementing epidemiological surveillance programs and continuous efforts to prevent and mitigate future outbreaks linked to emergent sequence types. It also underscores the significance of implementing proactive measures, robust risk assessment frameworks, and actively engaging the public to effectively address and minimize these risks.

## Methods

### Phylogenetic analysis

The genome sequences and metadata of 275 ST213, four ST313 and three ST34 *S*. Typhimurium strains were retrieved from the EnteroBase: *Salmonella* database (the assemblies met the quality control determined by the database: 4 Mbp–5.8 Mbp, >20 kb N50 value and <600 contigs)^3,14^, 270 of which were draft genome sequences and 5 were whole-genome sequences from ST213 strains. The sample includes 275 ST213 strains collected between 1957 and 2022 from 13 countries, Australia (2), Belgium (1), Canada (22), Denmark (5), France (2), India (3), Mexico (61), Netherlands (1), Portugal (2), Thailand (6), United Kingdom (71), USA (95) and Vietnam (4). These include 161 isolates from humans, 37 from animals, 11 from food, 1 from plants, 16 from water/rivers and 49 from an unknown source. Of these strains 43 were collected in Mexico between 2002 and 2005 during an integrated food chain surveillance program^8^.

Additionally, 5 whole-genome sequences of ST19, one ST313 and two ST34 *S*. Typhimurium strains were retrieved from the NCBI GenBank database^54^. Detailed information on the genomes metadata used in this study is available in Supplementary Table 1.

To build an SNP-based phylogeny, the 290 genomes were mapped to the *S*. Typhimurium genome SL1344 (accession no. FQ312003.1), which is a widely used reference strain in *S*. Typhimurium research. The SNPs were mapped, called, and filtered using the PhaME pipeline (v1.0.2)^55^. To build the phylogenetic tree of the core genome SNPs alignment (10208 bases), the best fitting substitution model out of 24 models was determined using MEGA (v11)^33^, the best fitting model for the maximum likelihood method was the Kimura 2-parameter model^56^. The tree was rooted at the reference genome node and the bootstrap method was used with 100 replications to assess the support of the ML phylogeny (bootstrapped tree: Supplementary Fig. 15A). Alternatively, a SNP maximum likelihood phylogeny was reconstructed in MEGA (v11) using the core genome alignment obtained from panaroo (v1.3.4) aligned using MAFFT (v7.520) and the SNPs extracted with SNP-sites (v.2.5.1) (Supplementary Fig. 15B). This was repeated for the plasmid phylogeny. To identify subpopulations FastBAPs (v1.0.8)^15^ was used under the following parameters: multi res baps with default prior and 2 levels. The clustering analysis was visualized using the ggtree (v3.4.0) package^57^. The analysis and visualization of the results were performed in RStudio (v2022.07.2 + 576) with R base version 4.2.1^58,59^.

A timed phylogeny was generated to explore the evolutionary history of the ST213 sequence type. 218 genomes with collection years were selected for the analysis and alignment using CSI phylogeny (v1.4)^60^ and a ML tree was constructed as described above, and the root-to-tip regression was calculated using TempEst v1.5.3^61^ (Supplementary Fig. 16). To estimate the timing of divergence of the lineages, the non-Bayesian RTDT-RelTime-ML method from MEGA (v11)^33,62^ was used with collection years as a constraint, the tree was rooted on S. Typhimurium LT2 (accession no. NC_003197.2), for confirmation LSD2 in IQTREE v2.0.3^63^ method was also used rendering similar results (Supplementary Fig. 17).

For the analysis of the CRISPR loci, the spacers were extracted using CRISPRCasFinder online^64^, then the galaxy platform was used to identify the spacers using the *Salmonella* CRISPR typing tool^65^. The spacers were added as metadata to an ML tree to confirm the geographical distribution of the isolates. Visualizations and annotations of all trees were made using iToL (v6)^66^.

### Accessory genome analysis (resistance, plasmids, virulence genes, phage content)

Genome assemblies of 281 isolates were examined for acquired AMR genes using the ResFinder database (v4)^67^. Plasmid replicon identification was performed using the PlasmidFinder database (v2.1)^68^ with default settings, an additional custom search was performed for the *repB* replicon for the D6-like plasmid prophage using MyDbFinder (v2.0)^69^. To further characterize the isolates the VFDB^31^ was used to determine the presence, absence, or interruption of virulence genes. Phage regions were identified using Phaster^70^. Heatmaps for all AMR genes and plasmid replicons by continent were created in RStudio (v2022.07.2 + 576) with R base version 4.2.1^58,59^ using the package ggplot2 (v3.3.6)^71^. The pseudogenes of the representative strains were extracted using the annotated data with the PGAP pipeline^72^ and were represented in a heatmap. The visualization of the phylogenetic trees with the metadata was made using iToL (v6)^66^.

### Bacterial strains

The strains used in the phenotypic analyses are listed in Supplementary Table 1. Representative strains were selected from M. B. Zaidi’s collection, which was built within the framework of an epidemiological surveillance. Only human isolates from invasive or severe gastrointestinal infections were chosen due to their relevance to public health, integrating strains from lineages 2 and 3. Among the selected strains the SLHS-03-15 strain was included, which unlike the rest of the strains does not contain an IncC-like plasmid. For all subsequent experiments, the strains were plated on LB agar (Luria-Bertani; 1% Bacto Tryptone, 0.5% yeast extract, 1% NaCl, and 1.5% Bacto agar) with 60 µg/ml chloramphenicol (ST213 strains except SLHS-03-15) or 50 µg/ml streptomycin (ST19 strains and SLHS-03-15) and incubated for 16 h at 37 °C. The pre-inoculums were prepared from single colonies of each strain resuspended in LB broth with the corresponding antibiotics and incubated at 37 °C with shaking (200 rpm) for 16–18 h. The mutant and complemented strains used in this study were generated by two step allelic exchange, as described previously^73^ and verified by PCR amplification and protein production, the strains and primers used are listed in Supplementary Table 2.

### RDAR morphotype assay

For the visualization of the RDAR morphotypes 20 µl of pre-inoculums of each strain were spotted in LB agar plates without NaCl, 40 µg/ml Congo red, and 20 µg/ml Coomassie blue R-250^74^. The plates were incubated at 20 °C in the dark. Every 5, 7, or 12 days, the plates were photographed. To analyze the macrocolonies a semi-quantitative approach using Fiji v2.35 software^75^ was performed, the image was split into channels and the red channel was used to count the wrinkles, rings, and area measurements. The appropriate thresholding for each image was set to distinguish the macrocolony base from the wrinkles and rings. To find categories between the strains, a hierarchical cluster analysis with the scaled numeric values was performed in RStudio (v2022.07.2 + 576) with R base version 4.2.1^58,59^. The experiments were performed in triplicate.

To quantify the Congo red bound from each macrocolony, the procedure described above was used with modifications, LB agar plates without NaCl and dyes were used. After incubation, the colonies were harvested and resuspended in sterile PBS. The suspension was centrifuged at 13,000 rpm for 3 min at room temperature, and the pellet was resuspended in 1 ml of 1 mg/ml Congo Red solution and incubated for 90 min with shaking (900 rpm) at room temperature. After the incubation, the samples were centrifuged at 13,000 rpm, to measure the OD_450_ of the supernatant, and 100 µl were transferred to a 96-well plate^76^ (GloMax®-Multi Detection System). To determine the concentration of the dye bound for each strain, a Congo red standard curve was used with limit values of 1 µg/ml to 1 mg/ml, using PBS as the diluent. Three independent biological replicates with technical duplicates were made. To summarize the results, a heatmap with scaled values was built using RStudio (v2022.07.2 + 576) with R base version 4.2.1 and the Tidyverse collection (v1.3.1)^58,59,77^.

A Pearson’s correlation analysis was constructed to find an association between the variables involved in the RDAR morphotype and the Congo red bound using RStudio (v2022.07.2 + 576) with the cor function in R base version 4.2.1 and the Corrplot package (v0.92)^58,59,78^.

### Biofilm formation

Biofilm formation was assessed using the crystal violet assay as described earlier^79^. The pre-inoculums of the strains were diluted 1:100 in LB broth without NaCl. In total, 100 µl of the dilution from each culture was transferred to a 96-well plate. Three individual 96-well plates were prepared for every biological replicate for evaluation at, 5, 7, and 12 days, and, incubated at 20 °C without shaking. After the incubation, the OD_600_ was measured, and the plates were washed three times with distilled water before the biofilms were fixed with methanol (200 µl) for 15 min and dried at room temperature for 10 min. For the staining, 200 µl of a crystal violet aqueous solution (0.2%) was added to each well for 10 min, the plate was washed three times with distilled water and dried. To quantify the biofilm formation, 125 µl of acid acetic aqueous solution (33%) was added for 15 min, and OD_560_ was measured. Three independent biological replicates with six technical replicates were performed. The biofilm formation was calculated as the ratio of OD_560_/OD_600_. To summarize the information, a heatmap with scaled values was built using RStudio (v2022.07.2 + 576) with R base version 4.2.1 and the Tidyverse collection (v1.3.1)^58,59,77^.

### Mammalian cell culture

Mouse embryonic fibroblasts (MEF) were maintained in 10 ml of Dulbecco′s Modified Eagle′s Medium (DMEM, Sigma-Aldrich) supplemented with 1 mM sodium pyruvate (Sigma-Aldrich), 1.5 g/l NaHCO_3_, and 10% v/v heat-inactivated Fetal Bovine Serum (Byproductos). C2BBe1 intestinal epithelial cells (ATCC^®^ CRL-2102™) were grown in 10 ml of DMEM Advanced (Gibco™) and supplemented with 200 mM L-glutamine (Sigma-Aldrich) and 10% (v/v) Fetal Bovine Serum. HT-29 (ATCC^®^ HTB-38™) intestinal epithelial cells were maintained in 10 ml of McCoy’s 5A medium (Sigma-Aldrich) supplemented with 1.5 g/l NaHCO_3_ and 10% v/v heat-inactivated Fetal Bovine Serum. THP-1 (ATCC^®^ TIB-2™) and U-937 (ATCC^®^ CRL-1593.2™) monocyte cell lines were maintained in Roswell Park Memorial Institute 1640 media (RPMI, Sigma-Aldrich) supplemented with 200 mM L-glutamine and 10% (v/v) heat-inactivated Fetal Bovine Serum.

One day before the experiments, MEFs, and C2BBe1 cells were seeded in 24-well plates at 2.6 × 10^6^ cells/ml per well^80,81^. Six to 7 days before the seeding in 24-well plates the HT-29 cells began to differentiate into a mixed population of enterocyte-like and columnar-like cells in DMEM depleted from glucose and supplemented with 10 mM galactose when the cells reached 2 × 10^5^ cells/ml, were seeded in 24-well plates at 3.9 × 10^6^ cells/ml per well and incubated for 25 days, changing the medium every 2 days^82^. Monocyte cell lines were seeded in 24-well plates at 6.5 × 10^6^ cells/ml per well and differentiated into macrophages with phorbol 12-myristate-12 acetate (PMA, Sigma-Aldrich). In total, 20 ng/ml of PMA was added to THP-1 cells and differentiated for 72 h, 12–18 h before the experiments, the media with PMA was replaced with fresh medium^83^. U-937 cells were differentiated with 30 ng/ml of PMA for 48 h, after the incubation, the medium was replaced with fresh medium and incubated for 5 days changing the medium every 2 days^84^.

The cell lines were incubated at 37 °C and 5% CO_2_. While growing, the cells were never allowed to reach above 9 × 10^5^ cells/ml. All the experiments were conducted with cell lines between passages 4–15, assuming as passage 1 the first subculture of the cell lines after being donated or purchased, disregarding the passage number indicated by the ATCC^®^.

### Bacterial infection of mammalian cells and LDH-release quantification

For aminoglycoside protection assays, 300 µl of pre-inoculum from each strain was diluted in 10 ml of LB broth without antibiotics in a 125 ml Erlenmeyer flask and incubated for 3.5 h at 37 °C with shaking (200 rpm). One ml of each bacterial culture was centrifuged at 8000 rpm for 2 min and the supernatant was discarded, the pellet was resuspended in 1 ml of PBS^85^. The infections of cell lines were conducted in 24-well plates treated as described above. All cell lines were infected at a multiplicity of infection (MOI) of 10 and incubated for 20 min at 37 °C and 5% CO_2_. After the incubation, the cells were washed three times with PBS and incubated with 500 µl of the corresponding cell culture media with 100 µg/ml gentamicin or amikacin (for the two isolates resistant to gentamicin). Invasion and replication measurements were conducted in separate wells. To quantify the invasion the cells were incubated 2 h in presence of the antibiotic to eliminate the extracellular bacteria and, for the replication measurements, the cells were incubated with 500 µl of the corresponding cell culture media with 10 µg/ml gentamicin or 25 µg/ml amikacin for 18 h. After the incubation periods, the wells were washed three times with PBS, and cell monolayers were incubated with 100 µl of Lysis solution (1% (v/v) Triton X-100 detergent (Bio-Rad), 0.1% (v/v) SDS (Bio-Rad) in PBS) for 3 min, finally 400 µl of PBS was added and the viable intracellular bacteria were enumerated. The invasion percentage was determined by dividing the CFU/ml at 2 h by the inoculum CFU/ml and, multiplying by 100. Similarly, the replication rate was calculated by dividing the CFU/ml at 18 h by the CFU/ml at 2 h^86^ (Supplementary Data).

For the adhesion assays, the cells were treated with 1 µg/ml cytochalasin D for 1 h. The monolayers were infected at a MOI 10 for 30 min and incubated at 37 °C and 5% CO_2_ after the incubation the cells were lysed, and the adherent bacteria enumerated. The adhesion percentage was calculated by dividing the CFU/ml at 30 min by the inoculum CFU/ml, multiplying the result by 100^87^. The experiments were performed with six independent biological replicates with technical duplicates unless otherwise is indicate.

LDH release of the cell cultures was quantified as an indirect measure of cytotoxicity using the CytoTox 96 Non-Radioactive kit (Promega) following the manufacturer’s instructions, the cell cultures were infected as described above and after 3 h p.i. and 21 h p.i. the supernatants were collected. Uninfected control cells were lysed with Triton X-100 detergent (Bio-Rad). The percentage of cytotoxicity was calculated as the quotient of the LDH release from the sample between the LDH release from uninfected cells multiplied by 100. The experiments were performed with three biological replicates with technical duplicates.

For the statistical analysis Kruskal–Wallis test followed by the Wilcoxon rank test was performed to identify differences between the strains. Statistical significance was determined to be *p* < 0.05. The graphs and the statistical analysis were built in RStudio (v2022.07.2 + 576) with R base version 4.2.1, the Tidyverse collection (v1.3.1), and the ggpubr package (v0.4.4)^58,59,77,88^.

### Motility assays

Bacterial pre-inoculums were adjusted to an OD_600_ = 2, 3 µl of the adjusted culture were spotted onto LB soft agar plates (0.3% Bacto agar), and incubated for 5 h at 37 °C, the plates were photographed, and the migration diameter was measured using FIJI software^75^, for the statistical analysis Kruskal–Wallis test was performed.

### Secreted and outer membrane protein profiles

For the secreted proteins, bacterial cultures were prepared from single colonies of each strain resuspended in LB broth with the corresponding antibiotics and incubated at 37 °C with shaking (200 rpm) for 13 h. The bacterial culture supernatants (3 ml) were recovered by centrifugation (12,000 rpm) and 320 µl of trichloroacetic acid was added and incubated for 1 h at 4 °C. The samples were centrifuged at 14,000 rpm for 30 min, and the supernatant was discarded. Each pellet was resuspended in 1 ml of acetone and then centrifuged at 12,000 rpm for 10 min, the acetone was removed, and the pellet was dried at room temperature for 5 min. Ten µl of 4X laemmli protein sample buffer and 2 µl of TRIS pH 11.1 was added to the samples.

For the outer membrane proteins, bacterial cultures were prepared from single colonies of each strain resuspended in LB broth without antibiotics and incubated overnight at 37 °C with shaking (200 rpm). In total, 1.5 ml of bacterial culture was concentrated by centrifugation (3 min at 12,000 rpm) and the supernatant was discarded. The pellet was resuspended in 1 ml of 10 mM Na_2_HPO_4_ (pH 7.2) and sonicated until the sample was clear, following by centrifugation for 2 min at 12,000 rpm. The supernatant was recovered and centrifuged for 30 min at 4 °C, then discarded, and the pellet was resuspended in 500 µl 10 mM Na_2_HPO_4_ (pH 7.2)/2% Triton X-100 and incubated for 30 min at 37 °C. The sample was centrifuged for 30 min at 12,000 rpm (4 °C), and the pellet was resuspended in 500 µl of 10 mM Na_2_HPO_4_ (pH 7.2), centrifuged for 30 min at 4 °C (12,000 rpm) and finally the pellet was resuspended in 50 µl of PBS. The proteins were quantified using the BCA method and 15 µg/µl was mixed with 10 µl of 4X laemmli protein sample buffer.

All the protein samples were heated for 10 min at 90 °C and analyzed by SDS-PAGE using 12% polyacrylamide gels and stained with Coomassie brilliant blue.

### Reporting summary

Further information on research design is available in the Nature Research Reporting Summary linked to this article.

## Supplementary information

Supplementary material

Dataset 1

Reporting Summary

## Data Availability

The data that support the findings are available as Supplementary Material. The files for the tree building, RDAR raw images, other raw data, and R scripts for the analysis and graphics are available upon request from the authors.
