# Supplementary material for: Population structure and ongoing microevolution of the emerging multidrug-resistant Salmonella Typhimurium ST213
Source: NPJ Antimicrob Resist. 2024 Apr 8;2:10. doi: 10.1038/s44259-024-00027-6 (PMC11721120; doi:10.1038/s44259-024-00027-6)
Supplement: Supplementary file 1 — Supplementary material [file 44259_2024_27_MOESM1_ESM.pdf]

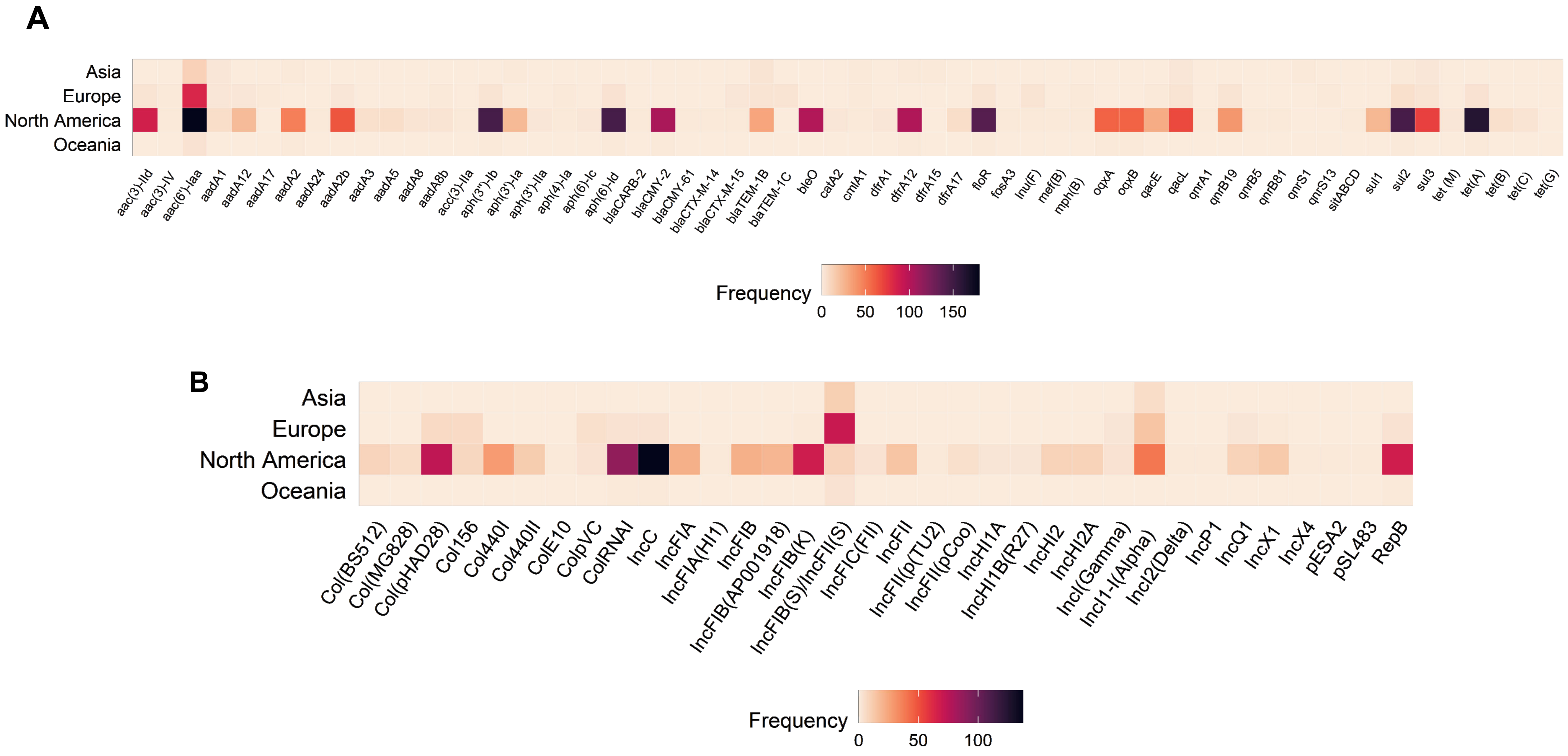

Supplementary Fig. 1| ST213 antimicrobial and plasmid content frequency by continent. A. Heatmap showing the frequencies of isolates with AMR determinants by continent. **B.** Heatmap displaying the frequencies of plasmid replicons in ST213 genotype. The visualizations were performed in Rstudio (for the information by strain see Supplementary Tables 4 and 5).

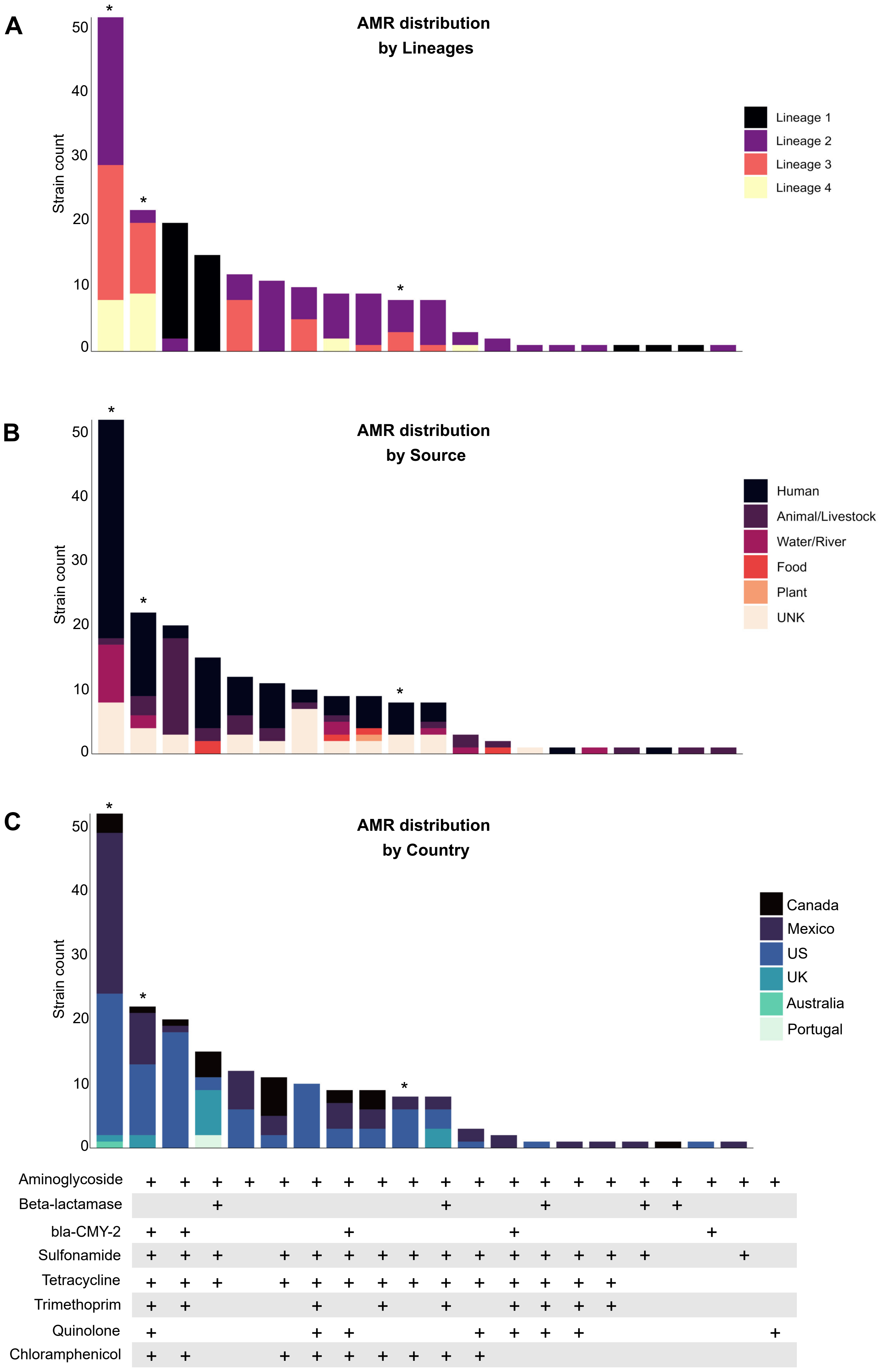
A
AMR distribution
by Lineages
Strain count
Lineage 1
Lineage 2
Lineage 3
Lineage 4
B
AMR distribution
by Source
Strain count
Human
Animal/Livestock
Water/River
Food
Plant
UNK
C
AMR distribution
by Country
Strain count
Canada
Mexico
US
UK
Australia
Portugal
Aminoglycoside
Beta-lactamase
bla-CMY-2
Sulfonamide
Tetracycline
Trimethoprim
Quinolone
Chloramphenicol

Supplementary Fig. 2| Distribution of AMR profiles by category. A. Number of strains with specific AMR gene content per lineage. **B.** Number of strains with specific AMR gene content per source. **C.** Number of strains with specific AMR gene content per country. * Multidrug-resistant profile (MDR)

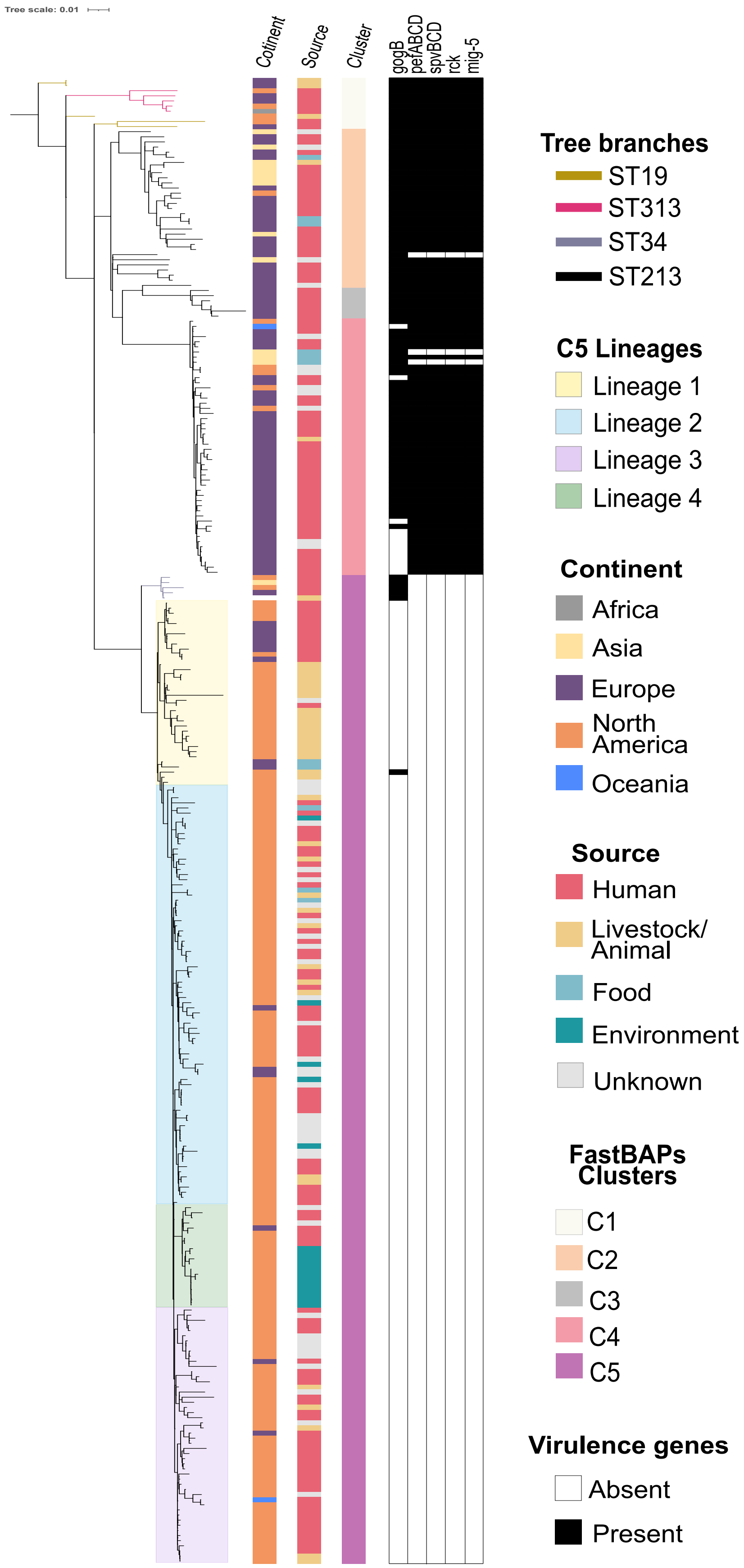

Supplementary Fig. 3| Presence/absence of relevant virulence genes in the ST213 genotype. ML tree of all the genomes used in the study coupled with relevant virulence genes present or absent in the ST213 genotype. The open squares indicate absence, and the filled squares indicate the presence of the gene. The virulence gene names are displayed on the top of the figure.

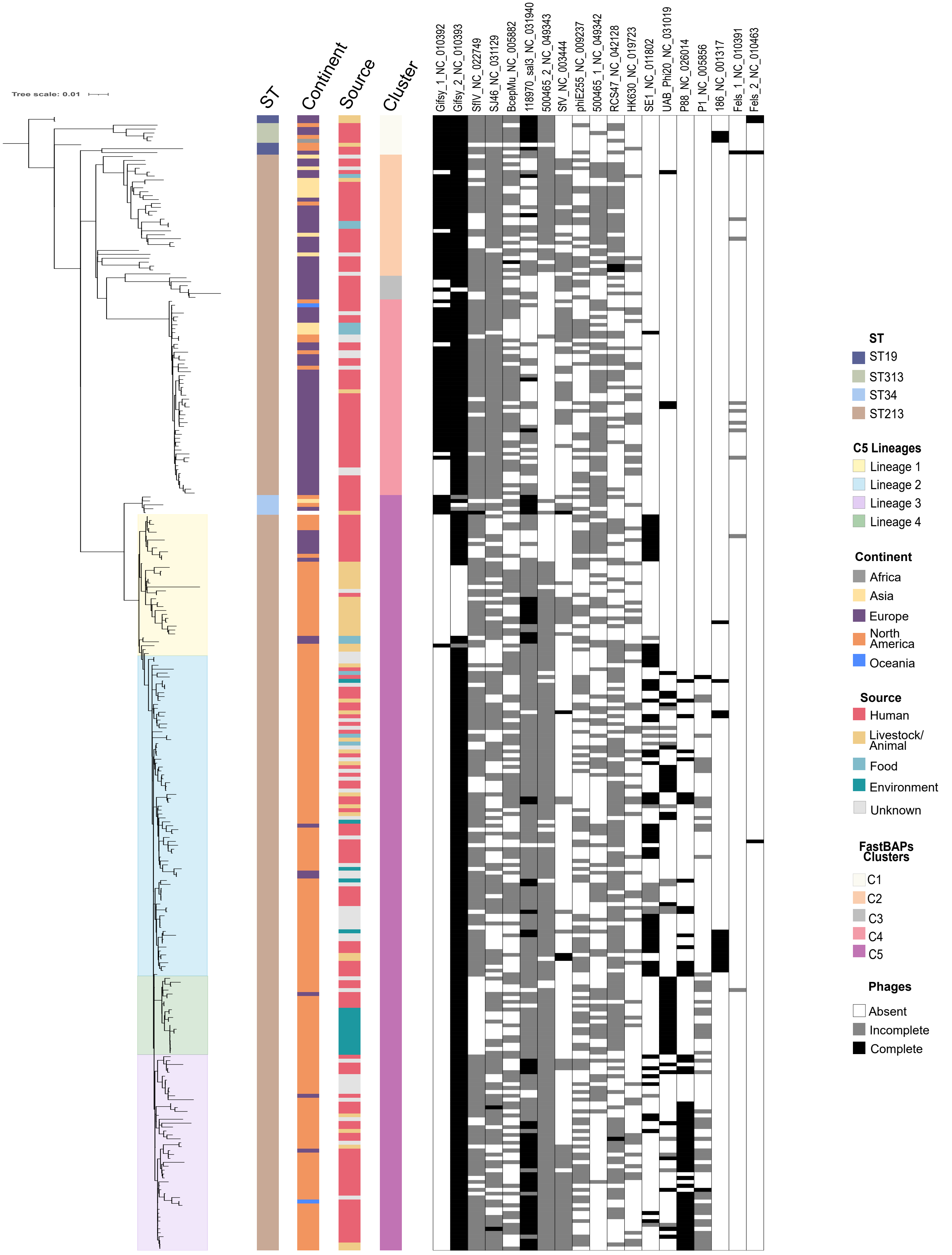

Supplementary Fig. 4| Prophage repertory of the ST213 genotype associated with the phylogeny. ML tree of all the genomes used in the study associated with the most abundant prophages found in the sample, present, absent and incomplete prophages are indicated for each strain (for complete prophage data see Supplementary Table 2).

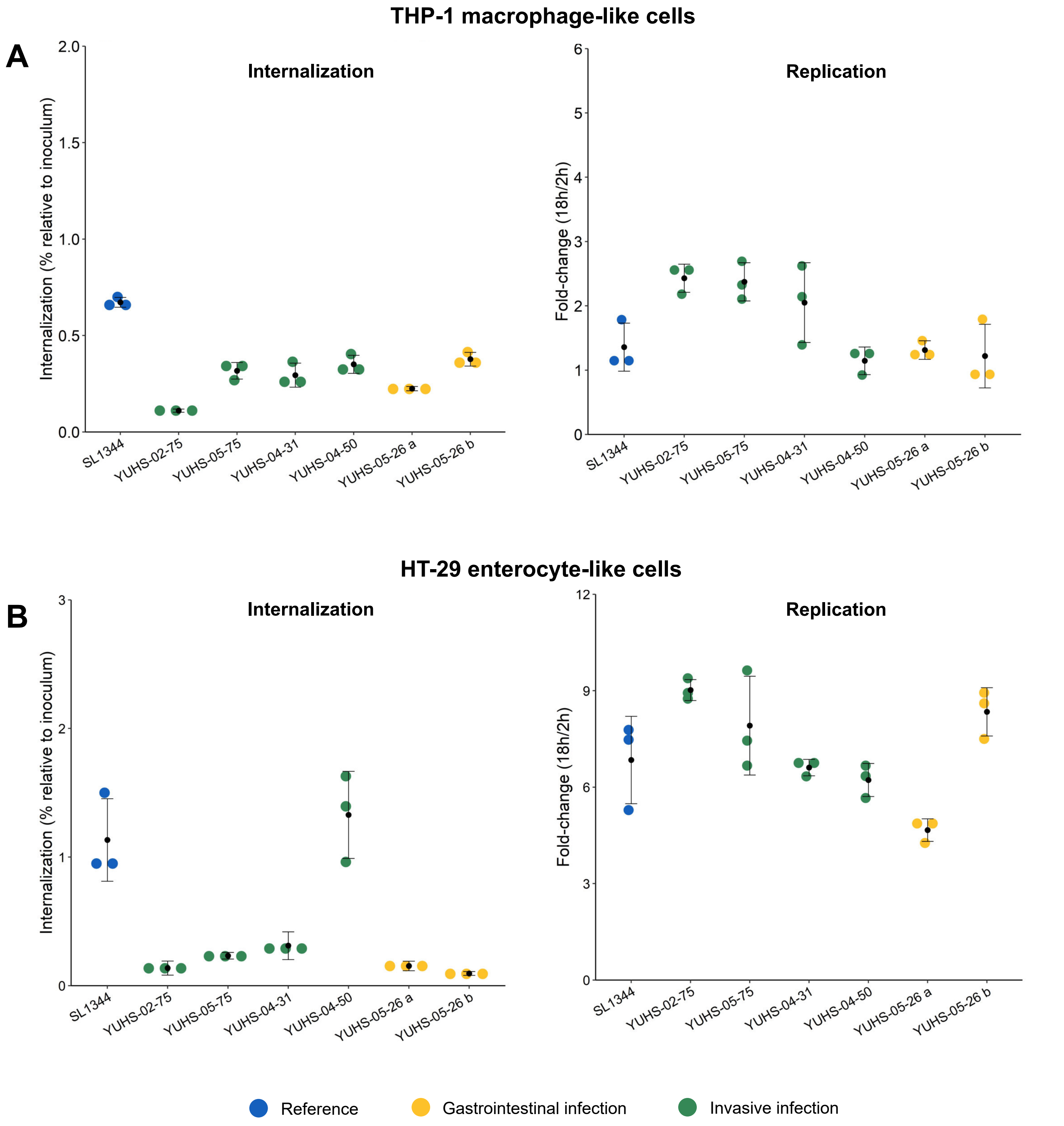

Supplementary Fig. 5| NA-ST213 internalization, and replication in eukaryotic cells. THP-1 (**A**) macrophage-like cells and, HT-29 (**B**) enterocyte-like cells were infected with NA-ST213 isolates from the Lineage 2. Internalization and replication were measured employing aminoglycoside protection assays, the cells were incubated with the bacterial culture for 2h (internalization) or 18h (replication), then lysed and counted (Colony-forming units/mL). Each dot represents an independent biological replicate (with two technical replicates), and the error bars indicate the standard deviation of n=3 replicates.

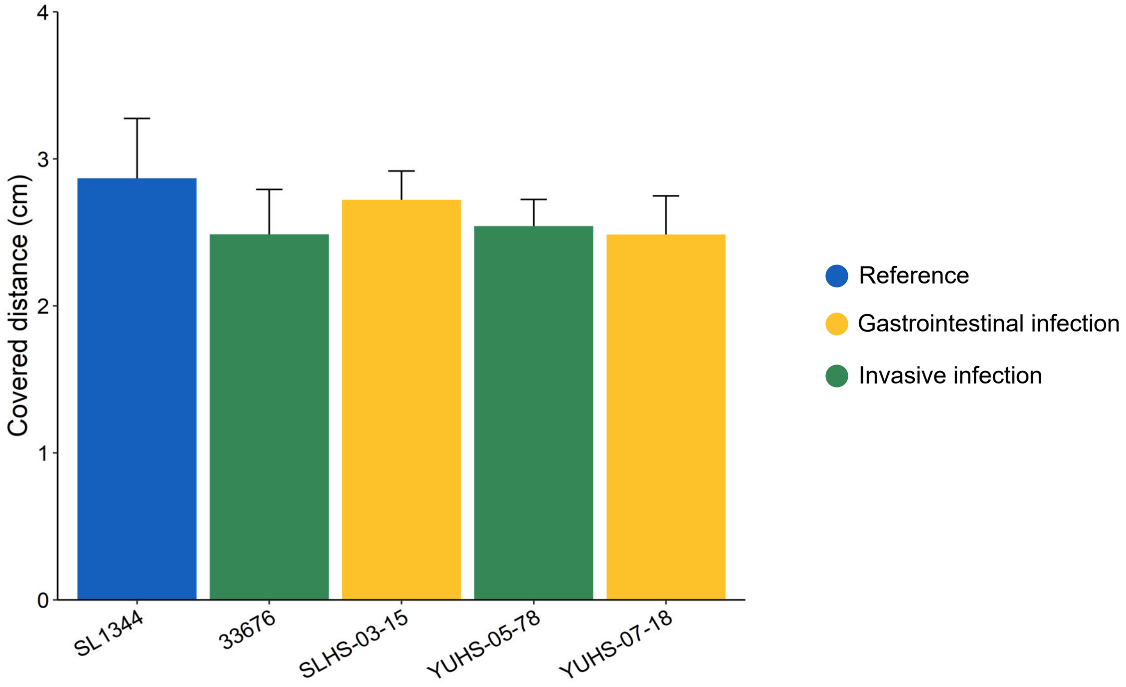

Supplementary Fig. 6| *S.* Typhimurium strains motility assay. S. Typhimurium strains were assayed on LB soft agar plates (0.3% Bacto agar) at 37°C. The plates were photographed at 5h, and, the migration diameter was measured using FIJI software.

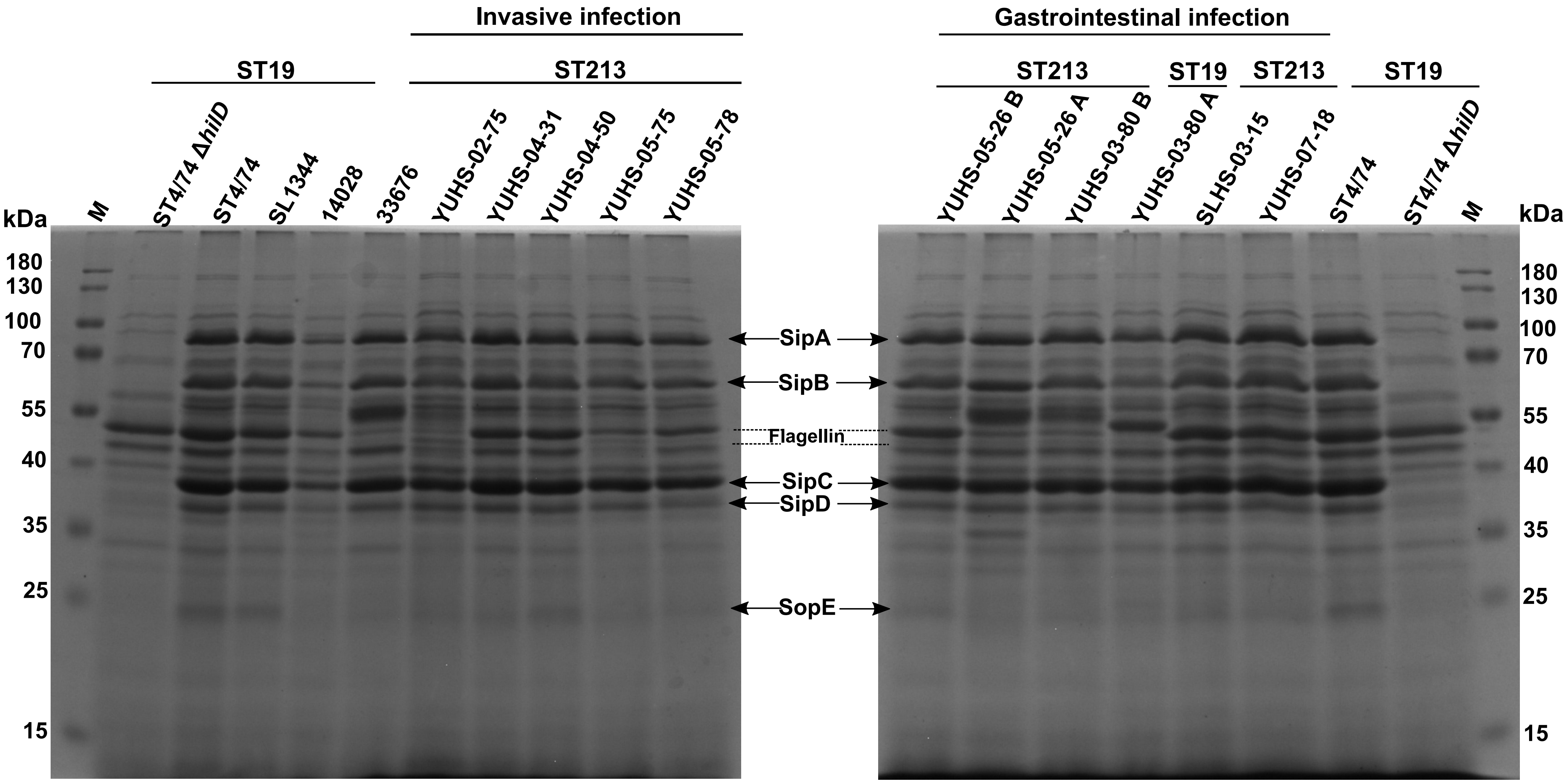

Supplementary Fig. 7| S. Typhimurium secreted proteins profiles. Secreted protein profiles of ST19 and ST213 WT strains and ST4/74 Δ*hilD* used as negative control Bacteria were growth in LB for 13h and the secreted proteins were recovered from the supernatants using trichloroacetic acid. The proteins were resolved by 12% SDS-PAGE and stained with Coomassie Brillant Blue.

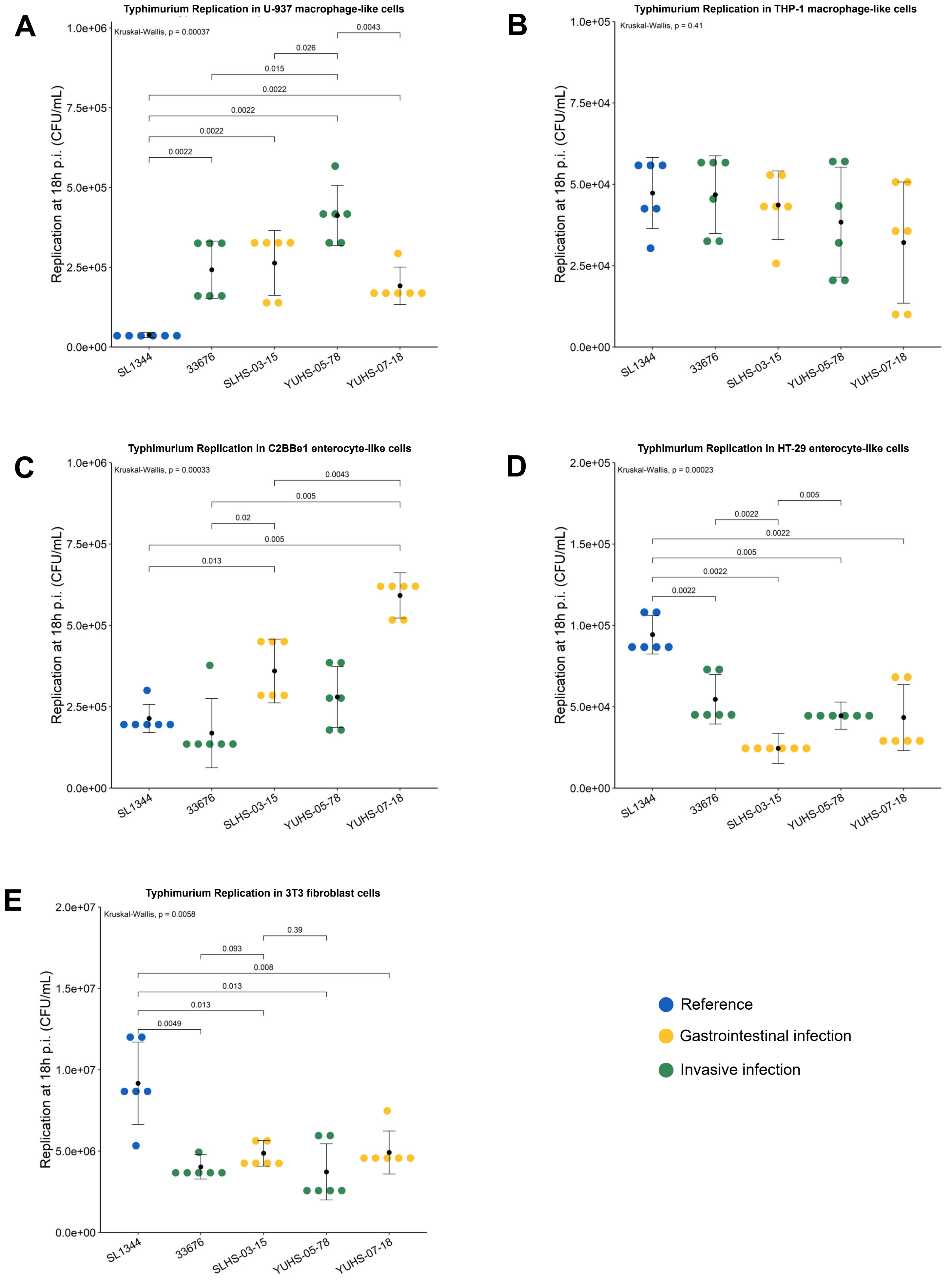

Supplementary Fig. 8| NA-ST213 replication in eukaryotic cells. U-937 (**A**), THP-1 (**B**) macrophage-like cells, C2BBe1 (**C**), HT-29 (**D**) enterocyte-like cells and, 3T3 fibroblasts (**E**) were infected with NA-ST213 isolates from the Lineage 2 and 3 and a reference strain. Replication were measured employing aminoglycoside protection assays, the cells were incubated with the bacterial culture for 18h, then lysed and counted (Colony-forming units/mL). Each dot represents an independent biological replicate (with two technical replicates), and the error bars indicate the standard deviation of n=6 replicates. This is the same data from Fig. 5, displayed as CFU/mL instead of as fold change.

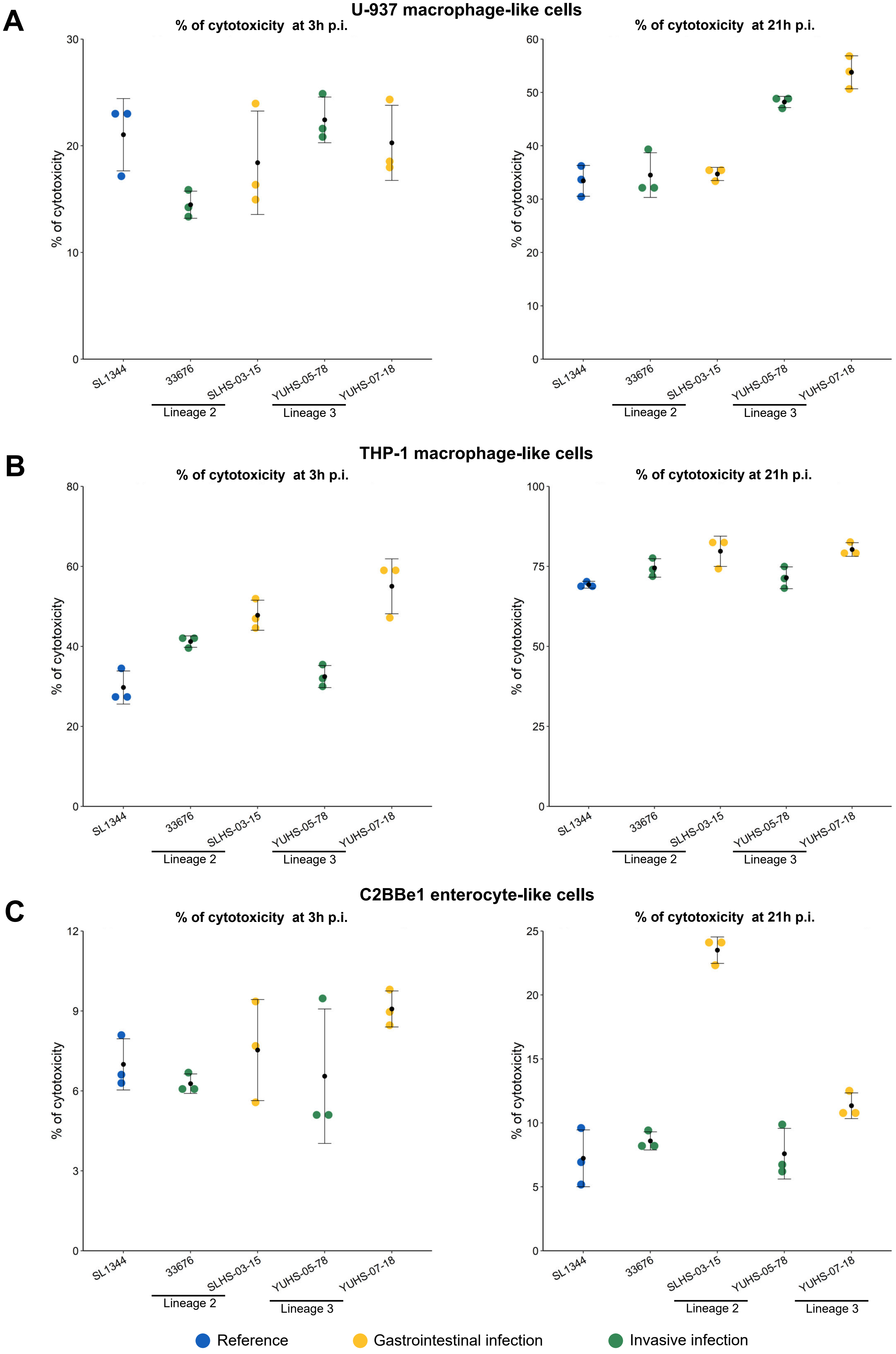

Supplementary Fig. 9| Cytotoxicity in eukaryotic cells infected with *S.* Typhimurium.
Legend continue in next page.

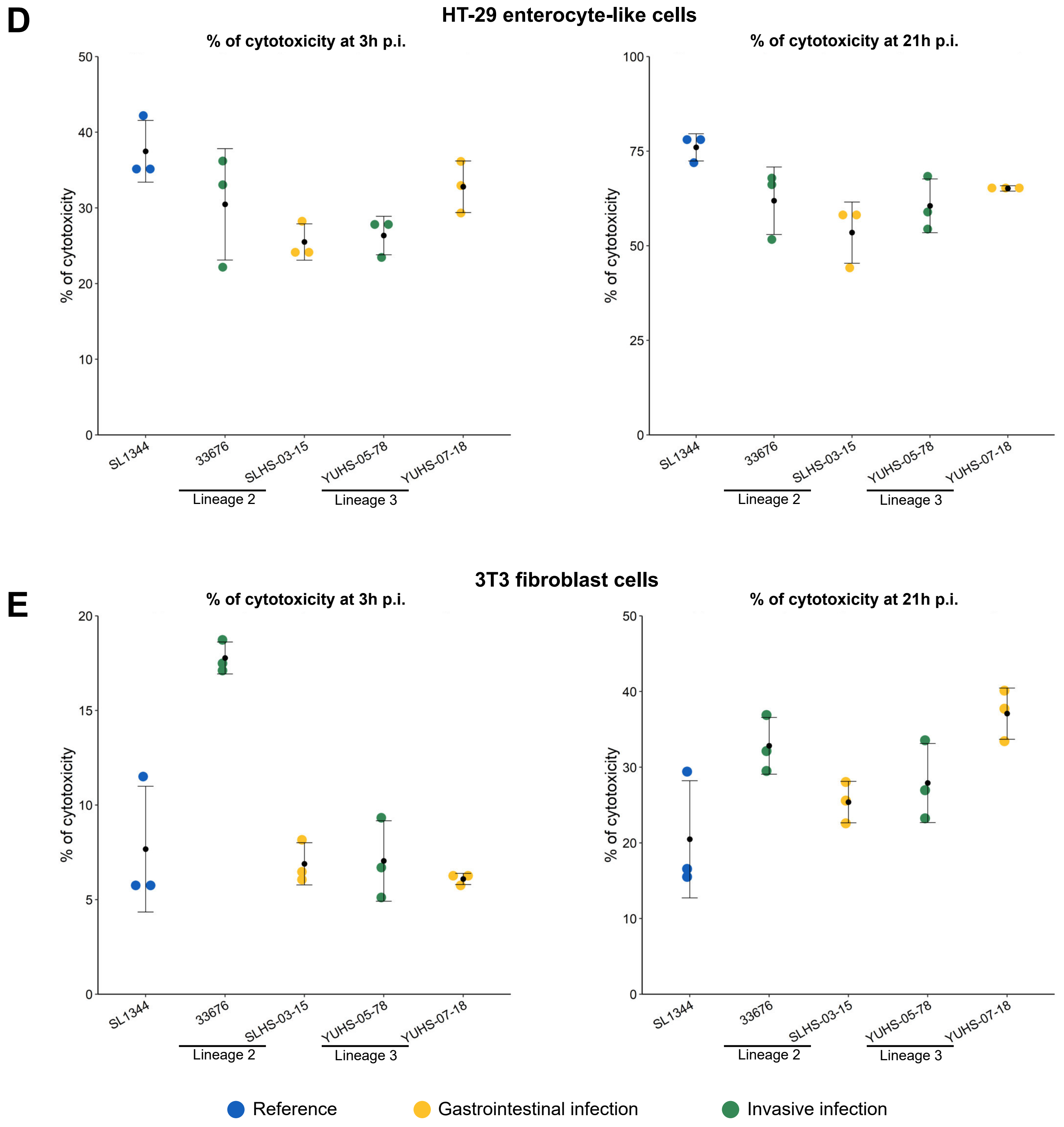

Supplementary Fig. 9| Cytotoxicity in eukaryotic cells infected with S. Typhimurium. U-937 (**A**), THP-1 (**B**) macrophage-like cells, C2BBe1 (**C**), HT-29 (**D**) enterocyte-like cells and, 3T3 fibroblasts (**E**) were infected with NA-ST213 isolates from the Lineage 2 and 3 and a reference strain. LDH release was quantified in the supernatants as an indirect measure of cytotoxicity at 3h and 21h post infection (p.i.). Each dot represents an independent biological replicate (with two technical replicates), and the error bars indicate the standard deviation of n=3 replicates.

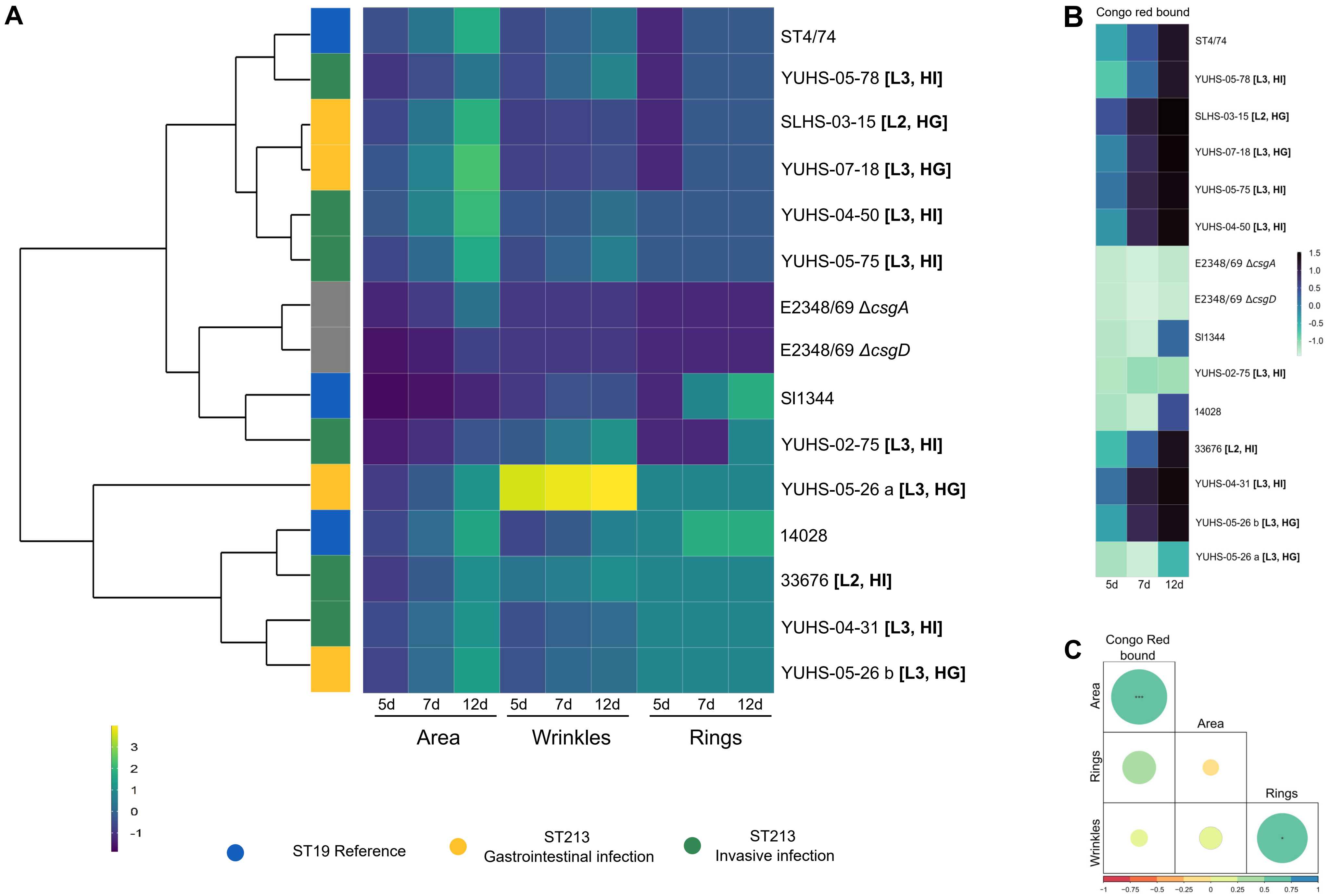

Supplementary Fig. 10| Clustering analysis of the RDAR morphotype characteristics. **A.** Images of the RDAR microcolonies were acquired at 5d, 7d, and 12d, the area was measured, and rings and wrinkles were counted using FIJI software. The clustering analysis of the scaled semiquantitative data was made in Rstudio. HG, gastrointestinal infection and HI, invasive infection. **B.** Heatmap displaying the concentration of Congo red binding by the RDAR macrocolonies through time. **C.** Correlation analysis between the architectural characteristics of the RDAR morphotype and Congo red binding, P-values are indicated (**P≤0.01, *** P≤0.001).

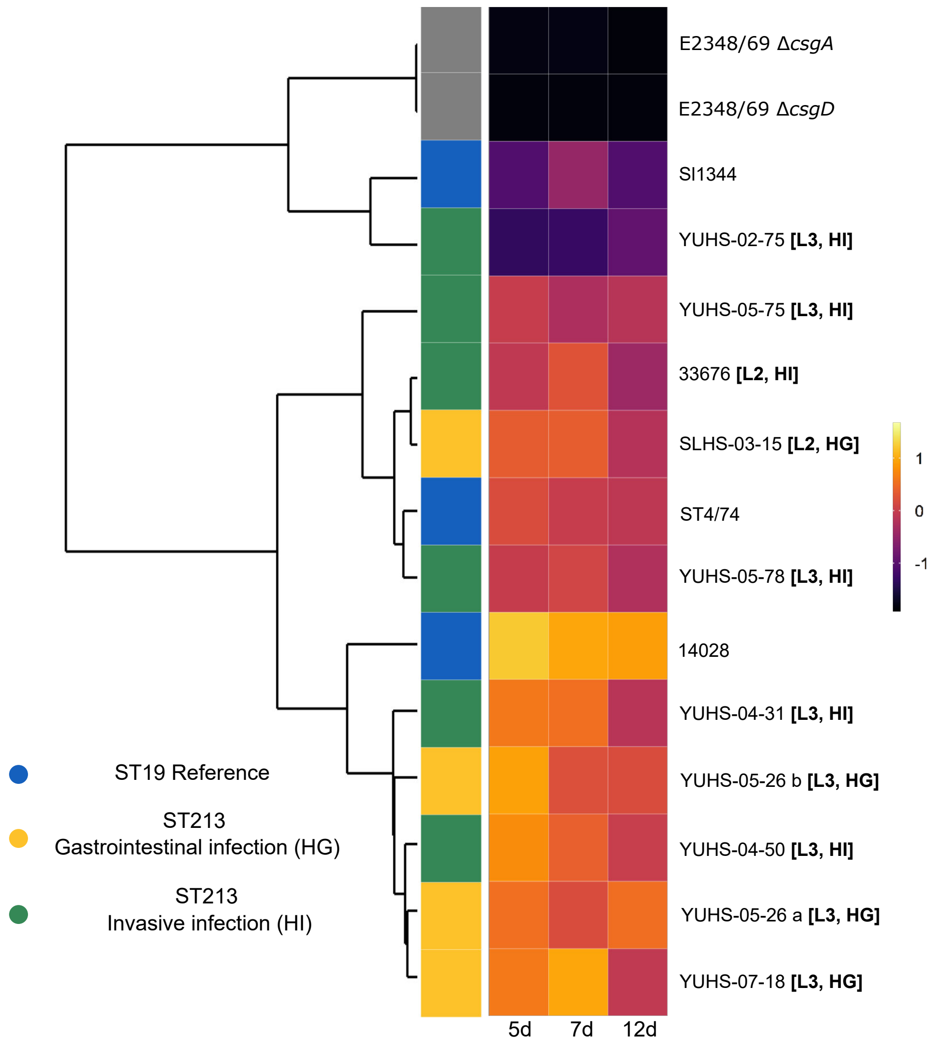

Supplementary Fig. 11| Clustering analysis of Biofilm formation by *S.* Typhimurium isolates. The strains ability to form biofilms at 20°C in LB without salt was eavluated at 5d, 7d, and 12d in microplates using the crystal violet assay. The acquired optical densities were procesed as descibed in the methods section. The clustering analysis of the scaled data was made in Rstudio.

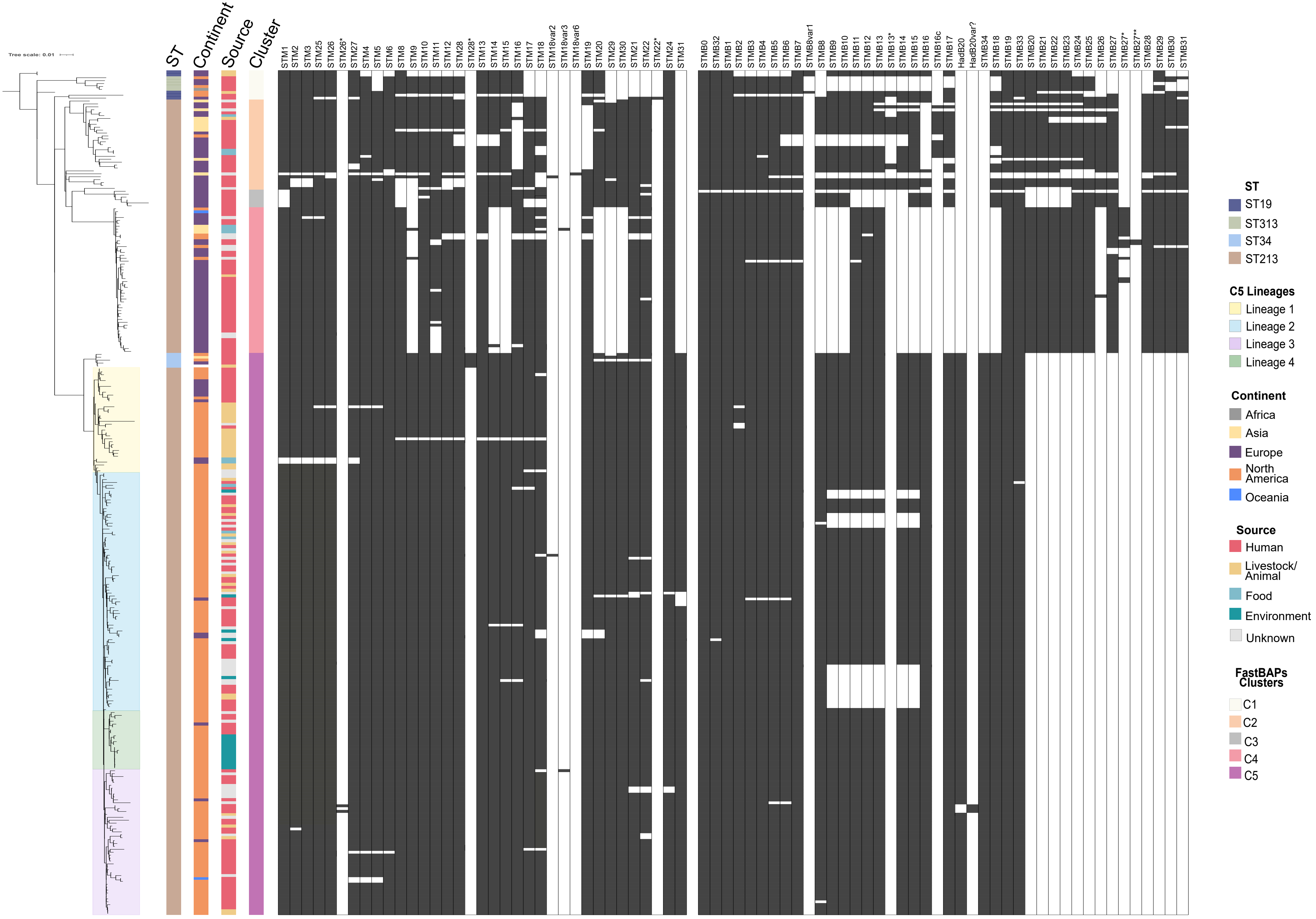

Supplementary Fig. 12| CRISPR spacer diversity of the ST213 genotype. ML tree coupled with spacer content in the two CRISPR loci; CRSPR1 and CRISPR2. The spacer names are displayed on the top, the open squares indicate absence and filled squares indicate presence of the spacer.

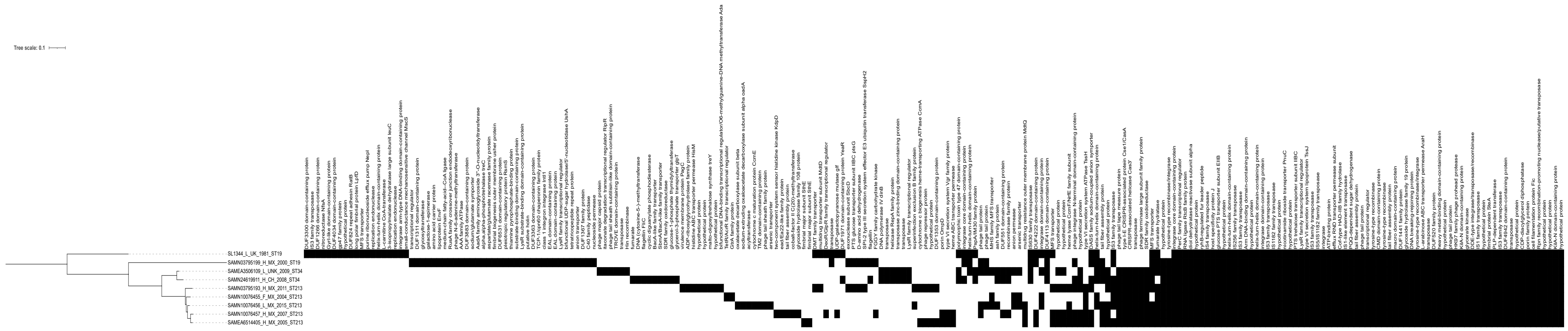

Supplementary Fig. 13| Putative pseudogenes identified in ST213, ST34 and ST19 reprentative strains. ML SNP tree coupled with the binary data of present (black) or absent (withe) putative pseudogenes. The names of the products are displayed on the top of the figure.

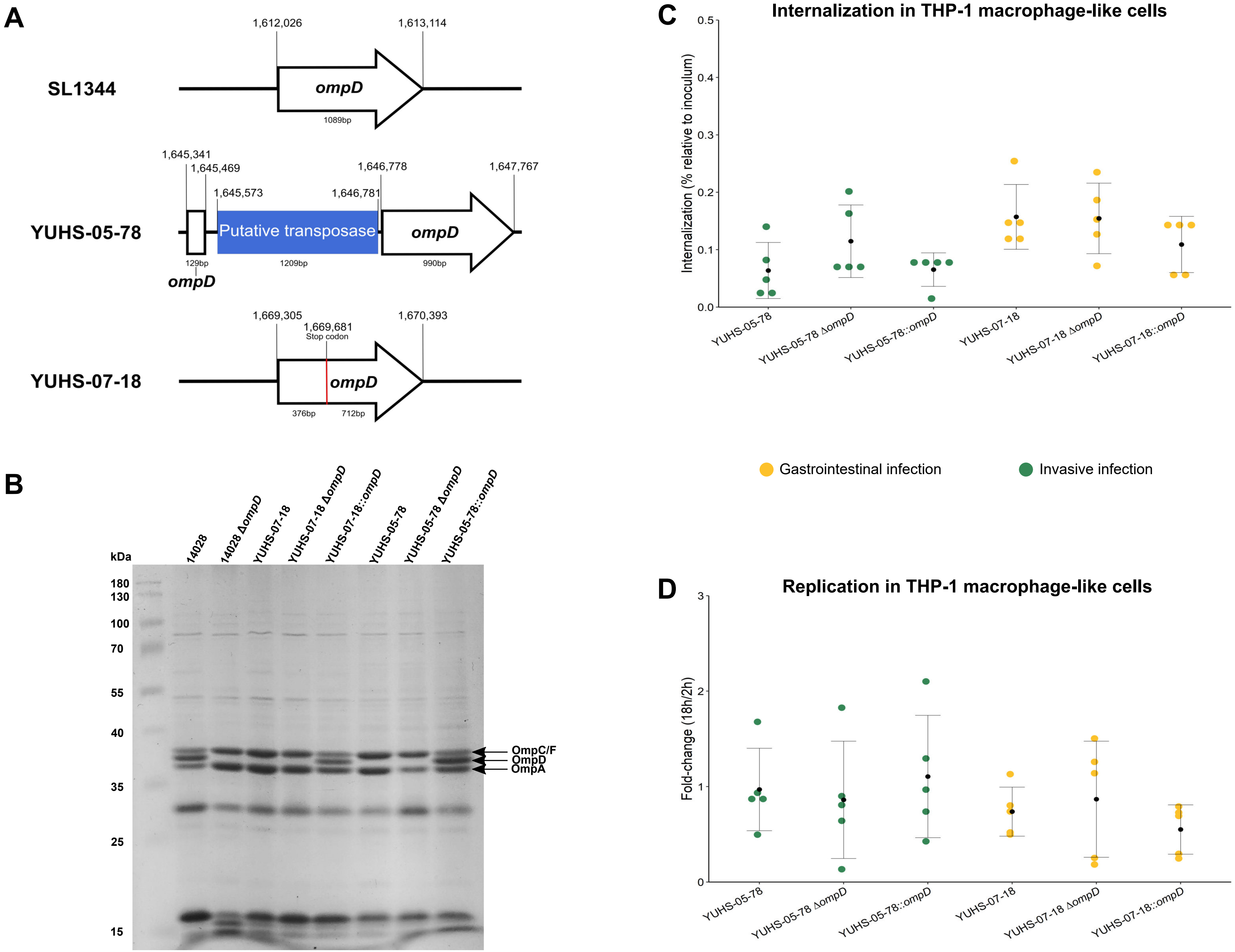
A
1,612,026
1,613,114
SL1344
ompD
1089bp
1,645,341
1,645,469
1,645,573
1,646,781
1,646,778
1,647,767
YUHS-05-78
Putative transposase
ompD
129bp
1209bp
990bp
ompD
1,669,305
1,669,681
Stop codon
1,670,393
YUHS-07-18
ompD
376bp
712bp
B
14028
14028 ΔompD
YUHS-07-18
YUHS-07-18 ΔompD
YUHS-07-18::ompD
YUHS-05-78
YUHS-05-78 ΔompD
YUHS-05-78::ompD
kDa
180
130
100
70
55
40
35
25
15
OmpC/F
OmpD
OmpA
C
Internalization in THP-1 macrophage-like cells
Internalization (% relative to inoculum)
0.5
0.4
0.3
0.2
0.1
0.0
YUHS-05-78
YUHS-05-78 ΔompD
YUHS-05-78::ompD
YUHS-07-18
YUHS-07-18 ΔompD
YUHS-07-18::ompD
Gastrointestinal infection
Invasive infection
D
Replication in THP-1 macrophage-like cells
Fold-change (18h/2h)
3
2
1
0
YUHS-05-78
YUHS-05-78 ΔompD
YUHS-05-78::ompD
YUHS-07-18
YUHS-07-18 ΔompD
YUHS-07-18::ompD

Supplementary Fig. 14| Two S. Typhimurium ST213 isolates show disruptions in the gene that encodes the immunogenic outer-membrane protein OmpD. (A) Schematic representation of the genomic disruptions in the two ST213 strains compared to the SL1344 reference strain, the genome coordinates of the gene are indicated on top of the diagram and the size of the gene or gene fragments are indicated below. **(B)** Typhimurium outer-membrane protein profiles of the selected wild-type, isogenic mutants and complemented strains showing the presence or absence of the OmpD protein. **(C)** Macrophage-like cells were infected with the two NA-ST213 strains and its isogenic *ompD* mutants. Internalization was measured using aminoglycoside protection assays, the cells were incubated with the bacterial culture for 2h then lysed and counted (Colony-forming units/mL). Each dot represents an independent biological replicate (with two technical replicates), and the error bars indicate the standard deviation of n=5 replicates. No significant differences were found between groups (for YUHS-05-78 group, Kruskal-wallis, p= 0.43; for YUHS-07-18 group, Kruskal-wallis, p= 0.38). **(D)** Macrophage-like cells were infected with the two NA-ST213 strains and its isogenic ompD mutants. Replication was measured using aminoglycoside protection assays, the cells were incubated with the bacterial culture for 18h then lysed and counted (Colony-forming units/mL). Each dot represents an independent biological replicate (with two technical replicates), and the error bars indicate the standard deviation of n=5 replicates. No significant differences were found between groups (for YUHS-05-78 group, Kruskal-wallis, p= 0.73; for YUHS-07-18 group, Kruskal-wallis, p= 0.53).

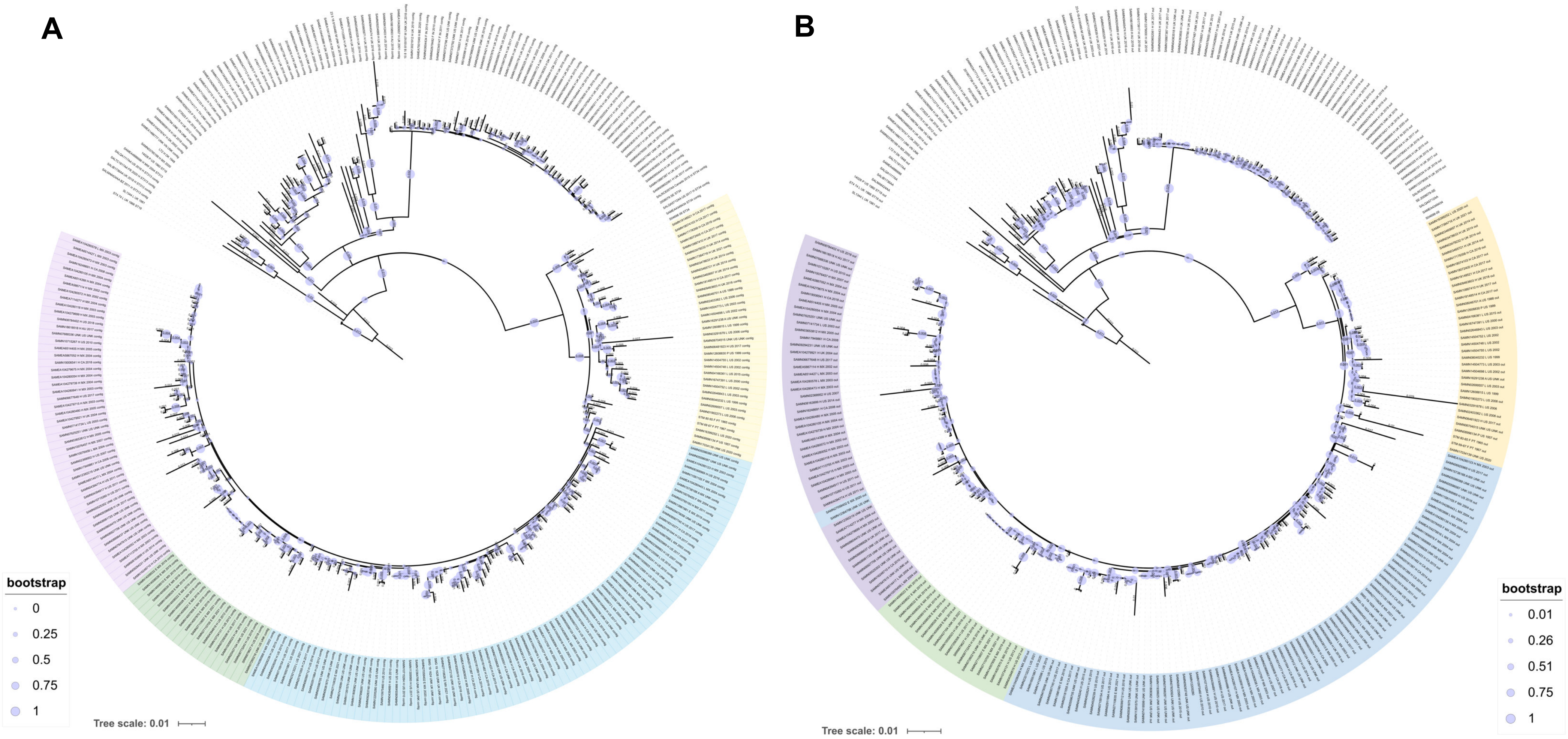

Supplementary Fig. 15| ML trees showing bootstraps and branch lengths. A. Tree showed in Figure 1, build using the PhaME pipeline and reconstructed in MEGA software. **B.** ML tree build using only the SNPs from the core genome, extracted using Panaroo, the tree was build in MEGA.

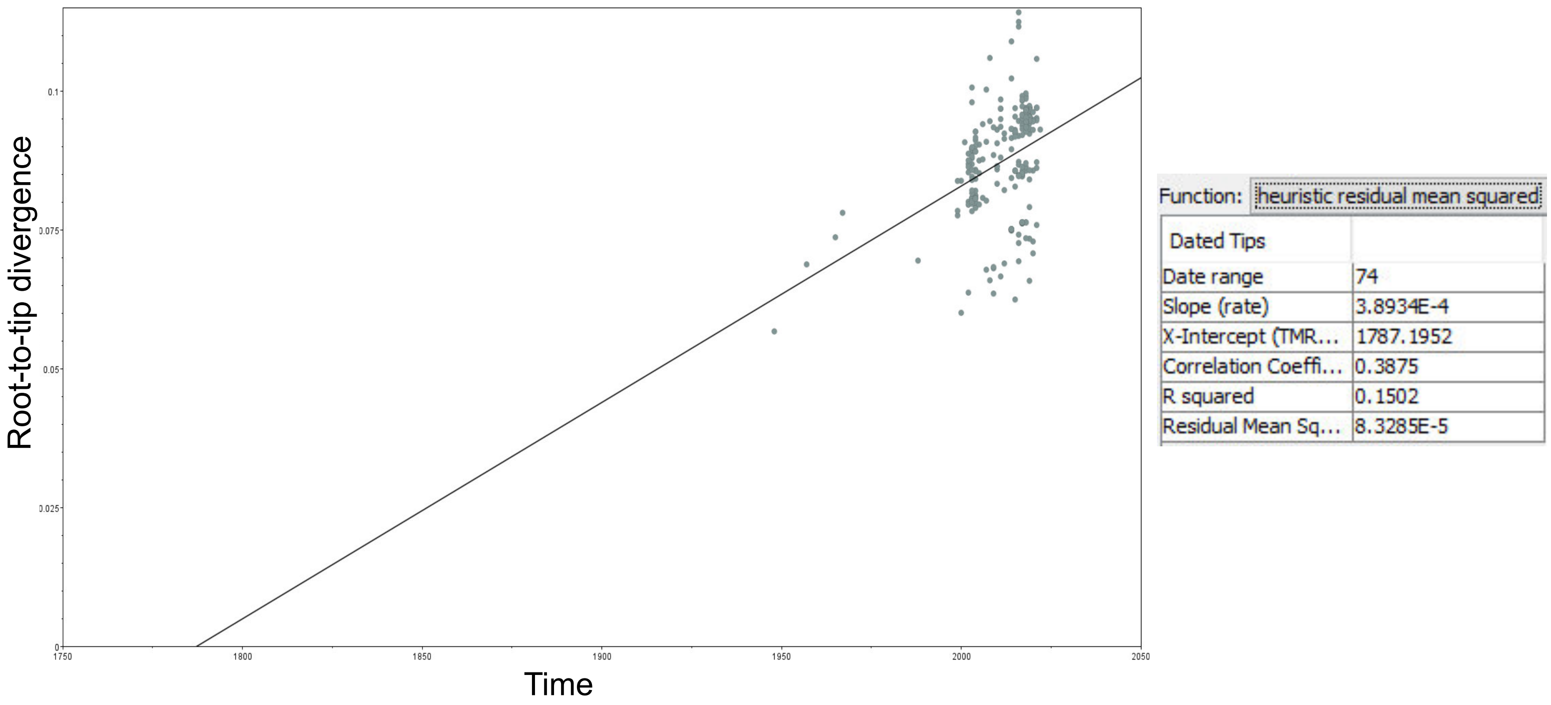

Supplementary Fig. 16| Root-to-tip regression of the ML tree used for Dating. Roort-to-Tip regression obtained using TempEst for the ML Tree used for dating with RelTime method in MEGA XI. or for dating using IQ-TREE v. 2.0.3 with the LSD2 method.

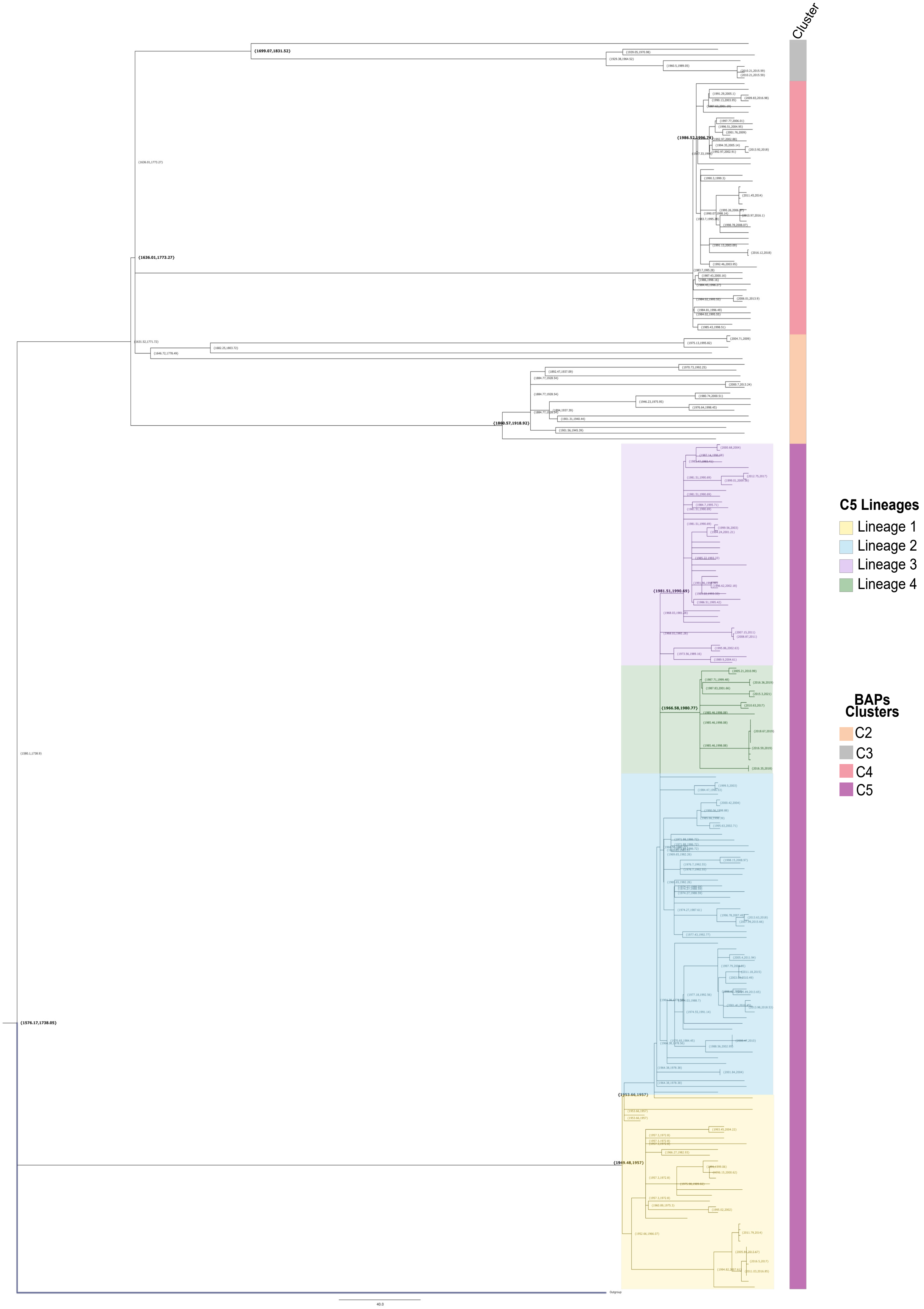

Supplementary Fig. 17| Dated phylogeny with LSD2 method. (legend in the next page)|

Supplementary Fig. 17| Dated phylogeny with LSD2 method. Dated phylogenetic with the LSD2 method implemented in IQ-TREE v2.0.3 tree of 218 (and outgroup) ST213 strains. Showing confidence intervals at every node, the more relevant nodes are indicated in bold.

Supplementary_Table_1

Key			
Country		Isolate Source	
MX	Mexico	H	Human
MW	Malawi	F	Food
UK	United Kingdom	P	Poultry
US	United States of America	L	Livestock
DK	Denmark	A	animal (companion or wild animals)
VN	Vietnam	E	Environmental (water/River/Plant)
TH	Thailand		
FR	France		
NL	Neatherlands		
CA	Canada		
PT	Portugal		
AU	Australia		
CH	China		
UNK	Unknown		

Strains in red are part of the Zaidi collection

Strain_label (SAMPLEIDENTEROBASE_Source_Country_CollectionDate)	Cluster	Lineage	Source	Country	Name in Zaidi collection
SALTC1917AA_H_FR_2020_ST313	1	NONE	Human	FR	
SALQA1117AA_H_US_2016_ST313	1	NONE	Human	US	
SALMA6424AA_H_BZ_2011_ST313	1	NONE	Human	BZ	

SALIB1736AA_H_UK_2018_ST313	1	NONE	Human	UK	
SALQA5712AA_H_US_2017_ST34	5	NONE	Human	US	
SALRC8357AA_H_CA_2015_ST34	5	NONE	Human	CA	
SAMEA4349434_H_DK_2017_ST34	5	NONE	Human	DK	
SAMEA3506109_L_UNK_2009_ST34	5	NONE	Livestock	UNK	
SAMN24619911_H_CH_2008_ST34	5	NONE	Human	CH	
SAMEA4886686_H_MW_2004_ST313	1	NONE	Human	MW	
ST4_74_L_UK_1966_ST19	1	NONE	Livestock	UK	
SL1344_L_UK_1981	1	NONE	Livestock	UK	
14028_P_US_1960_ST19	1	NONE	Poultry	US	
SAMN03795199_H_MX_2000_ST19	1	NONE	Human	MX	
LT2_H_DK_1948_ST19	1	NONE	Human	DK	
SAMEA1486777_UNK_VN_UNK	2	NONE	UNK	VN	
SAMEA104279747_H_DK_2002	2	NONE	Human	TH	
SAMEA7113715_H_TH_UNK	2	NONE	Human	TH	
SAMEA104280564_H_UK_2007	2	NONE	Human	UK	
SAMEA7113595_H_TH_UNK	2	NONE	Human	TH	
SAMEA7114133_H_TH_UNK	2	NONE	Human	TH	
SAMEA2708406_H_VN_UNK	2	NONE	Human	VN	
SAMN09009175_H_UK_2016	2	NONE	Human	UK	
201907738_H_FR_UNK	2	NONE	Human	FR	
201902876_H_FR_UNK	2	NONE	Human	FR	
SAMN03477712_H_UK_2014	2	NONE	Human	UK	
SAMN11043670_F_UK_2016	2	NONE	Food	UK	
474917_F_UK_2016	2	NONE	Food	UK	
SAMN03466029_H_UK_2014	2	NONE	Human	UK	
SAMEA1486794_UNK_VN_UNK	2	NONE	UNK	VN	
SAMN03169175_H_UK_2012	2	NONE	Human	UK	
273525_F_UK_2016	2	NONE	Food	UK	
SAMN09009119_H_UK_2018	2	NONE	Human	UK	
SAMN18292725_P_TH_2019	2	NONE	Poultry	TH	
SAMEA7114117_H_TH_UNK	2	NONE	Human	TH	
SAMN12324696_H_UK_2019	2	NONE	Human	UK	
SAMEA7113668_H_NL_2009	2	NONE	Human	NL	

SAMN17727212_H_CA_2011	2	NONE	Human	CA	
SAMN13435427_H_UK_2019	2	NONE	Human	UK	
SAMEA1486844_UNK_VN_UNK	2	NONE	UNK	VN	
SAMN03479844_H_UK_2015	2	NONE	Human	UK	
SAMEA104280632_H_UK_2000	2	NONE	Human	UK	
SAMEA6514117_H_DK_2008	2	NONE	Human	DK	
SAMEA104403886_H_DK_2009	2	NONE	Human	DK	
SAMEA104138383_H_DK_2009	2	NONE	Human	DK	
23-5-18-8100480-M_UNK_UK_2018	2	NONE	UNK	UK	
SAMN21925839_H_UK_2021	3	NONE	Human	UK	
SAMEA7112595_H_UK_2003	3	NONE	Human	UK	
SAMN03480329_H_UK_2014	3	NONE	Human	UK	
SAMN20665971_H_UK_2016	3	NONE	Human	UK	
SAMN09445474_H_UK_2016	3	NONE	Human	UK	
SAMN09204889_H_UK_2016	3	NONE	Human	UK	
SAMN07695344_H_UK_2015	4	NONE	Human	UK	
SAMN13379000_H_UK_2019	4	NONE	Human	UK	
SAMN10173917_H_UK_2018	4	NONE	Human	UK	
SAMN09652061_H_UK_2017	4	NONE	Human	UK	
SAMN10887387_H_UK_2017	4	NONE	Human	UK	
SAMN09504443_H_UK_2017	4	NONE	Human	UK	
SAMN04363518_H_UK_UNK	4	NONE	Human	UK	
SAMN03475790_H_UK_2014	4	NONE	Human	UK	
SAMN04363855_H_UK_UNK	4	NONE	Human	UK	
SAMN04576500_UNK_UK_2015	4	NONE	UNK	UK	
SAMN07155657_H_UK_2017	4	NONE	Human	UK	
SAMN03477487_UNK_UK_2014	4	NONE	Human	UK	
SAMEA104280657_H_UK_2001	4	NONE	Human	UK	
SAMN04600446_H_UK_2015	4	NONE	Human	UK	
SAMN10076176_H_UK_2018	4	NONE	Human	UK	
SAMN08412403_H_US_2014	4	NONE	Human	US	
SAMN10094178_H_UK_2018	4	NONE	Human	UK	
SAMN10080211_H_UK_2018	4	NONE	Human	UK	
SAMN07998294_UNK_US_UNK	4	NONE	UNK	US	

SAMN13869895_H_UK_2020	4	NONE	Human	UK	
SAMN03654434_F_IN_2015	4	NONE	Food	IN	
SAMN18619893_H_AU_2018	4	NONE	Human	AU	
10-8-18-8100167-W_UNK_UK_2018	4	NONE	UNK	UK	
SAMN11644844_H_UK_2019	4	NONE	Human	UK	
SAMN09257879_H_UK_2018	4	NONE	Human	UK	
SAMN20606976_UNK_UK_2018	4	NONE	UNK	UK	
SAMN09516421_H_UK_2018	4	NONE	Human	UK	
SAMN13379055_H_UK_2019	4	NONE	Human	UK	
SAMN13502534_H_UK_2019	4	NONE	Human	UK	
SAMN09468131_H_UK_2017	4	NONE	Human	UK	
SAMN10907354_H_UK_2017	4	NONE	Human	UK	
SAMN13379043_H_UK_2019	4	NONE	Human	UK	
SAMN07714455_H_UK_2015	4	NONE	Human	UK	
SAMN07690325_H_UK_2015	4	NONE	Human	UK	
SAMN26659974_UNK_US_2022	4	NONE	UNK	US	
SAMN02345147_F_IN_2011	4	NONE	Food	IN	
MS160098_H_UK_2016	4	NONE	Human	UK	
SAMN13704663_H_UK_2019	4	NONE	Human	UK	
SAMN02678482_F_IN_2010	4	NONE	Food	IN	
SAMN07372796_UNK_US_UNK	4	NONE	UNK	US	
SAMN07372792_UNK_US_UNK	4	NONE	UNK	US	
SAMN12009574_H_UK_2019	4	NONE	Human	UK	
SAMN14068083_H_UK_2020	4	NONE	Human	UK	
SAMEA7501049_H_BE_2020	4	NONE	Human	BE	
SAMEA104138353_H_DK_2011	4	NONE	Human	DK	
SAMN25686172_A_UK_2009	4	NONE	Animal	UK	
SAMN13021912_H_UK_2019	4	NONE	Human	UK	
SAMEA4983944_H_DK_2017	4	NONE	Human	DK	
SAMN10140066_H_UK_2018	4	NONE	Human	UK	
SAMN10058699_H_UK_2018	4	NONE	Human	UK	
SAMN17384716_H_UK_2021	5	Lineage 1	Human	UK	
SAMN03465721_H_UK_2014	5	Lineage 1	Human	UK	
SAMN03465697_H_UK_2014	5	Lineage 1	Human	UK	

SAMN03478633_H_UK_2014	5	Lineage 1	Human	UK	
SAMN03478232_H_UK_2014	5	Lineage 1	Human	UK	
SAMN17178358_H_CA_2018	5	Lineage 1	Human	CA	
SAMN09463603_H_UK_2018	5	Lineage 1	Human	UK	
SAMN18074103_H_CA_2017	5	Lineage 1	Human	CA	
SAMN10887410_H_UK_2017	5	Lineage 1	Human	UK	
SAMN18072405_H_CA_2017	5	Lineage 1	Human	CA	
SAMN18148521_H_CA_2017	5	Lineage 1	Human	CA	
SAMN18148514_H_CA_2017	5	Lineage 1	Human	CA	
SAMN08046701_A_US_1988	5	Lineage 1	Animal	US	
SAMN04166361_L_US_2015	5	Lineage 1	Livestock	US	
SAMN12608830_P_US_1999	5	Lineage 1	Poultry	US	
SAMN16747391_L_US_2000	5	Lineage 1	Livestock	US	
SAMN02646843_L_US_2003	5	Lineage 1	Livestock	US	
SAMN14504752_L_US_2002	5	Lineage 1	Livestock	US	
SAMN14504755_L_US_2002	5	Lineage 1	Livestock	US	
SAMN14504748_L_US_2002	5	Lineage 1	Livestock	US	
SAMN08040232_L_US_1999	5	Lineage 1	Livestock	US	
SAMN14504773_L_US_2003	5	Lineage 1	Livestock	US	
SAMN14504698_L_US_2002	5	Lineage 1	Livestock	US	
SAMN02699557_L_US_2003	5	Lineage 1	Livestock	US	
SAMN16291238_A_US_UNK	5	Lineage 1	Animal	US	
SAMN12608815_L_US_1999	5	Lineage 1	Livestock	US	
SAMN03291678_L_US_2006	5	Lineage 1	Livestock	US	
SAMN01902273_L_US_2006	5	Lineage 1	Livestock	US	
SAMN02403362_L_US_2006	5	Lineage 1	Livestock	US	
SAMN06704515_UNK_US_UNK	5	Lineage 1	UNK	US	
SAMN06481923_H_US_2017	5	Lineage 1	Human	US	
SAMN16399252_L_US_2020	5	Lineage 1	Livestock	US	
SAMN08996134_P_US_1957	5	Lineage 1	Poultry	US	
STM_80-65_F_PT_1965	5	Lineage 1	Food	PT	
STM_69-67_F_PT_1967	5	Lineage 1	Food	PT	
SAMN17034139_UNK_US_2020	5	Lineage 1	UNK	US	
SAMEA104280123_H_MX_2003	5	Lineage 2	Human	MX	SLHS03-15

SAMN05596088_UNK_US_UNK	5	Lineage 2	UNK	US	
SAMN05596087_UNK_US_UNK	5	Lineage 2	UNK	US	
SAMN15738188_A_MX_UNK	5	Lineage 2	Animal	MX	
SAMN06925969_H_US_2017	5	Lineage 2	Human	US	
SAMN06368969_H_US_2016	5	Lineage 2	Human	US	
SAMN11581155_F_MX_2004	5	Lineage 2	Food	MX	MIRES04-5
SAMEA104280443_L_MX_2004	5	Lineage 2	Livestock	MX	MIRES04-4
SAMN11803898_L_MX_2004	5	Lineage 2	Livestock	MX	SORAPUS04-16
SAMEA104280139_H_MX_2004	5	Lineage 2	Human	MX	SOHS04-19
SAMN11806968_UNK_MX_2004	5	Lineage 2	UNK	MX	SORAPUS04-23
SAMN11806976_F_MX_2004	5	Lineage 2	Food	MX	SOPUS04-20-2
SAMN10076455_F_MX_2004	5	Lineage 2	Food	MX	SO21
SAMEA104279916_L_MX_2004	5	Lineage 2	Livestock	MX	SORES04-45
SAMN08865452_UNK_US_UNK	5	Lineage 2	UNK	US	
SAMN07258826_UNK_US_UNK	5	Lineage 2	UNK	US	
SAMN20181423_H_CA_2015	5	Lineage 2	Human	CA	
SAMN20181482_H_CA_2016	5	Lineage 2	Human	CA	
SAMN20181389_H_CA_2014	5	Lineage 2	Human	CA	
SAMN20181387_H_CA_2014	5	Lineage 2	Human	CA	
SAMEA104280932_H_MX_2005	5	Lineage 2	Human	MX	MIHS05-11A
SAMN03795193_H_MX_2011	5	Lineage 2	Human	MX	33676
SAMN09213256_UNK_US_UNK	5	Lineage 2	UNK	US	
SAMN18350919_H_CA_2018	5	Lineage 2	Human	CA	
SAMN08472130_UNK_US_UNK	5	Lineage 2	UNK	US	
SAMN27110626_E_MX_2021	5	Lineage 2	Water/River	MX	
SMG-19-1626_UNK_UK_UNK	5	Lineage 2	UNK	UK	
SMG-19-1624_UNK_UK_UNK	5	Lineage 2	UNK	UK	
SAMN11806972_L_MX_2004	5	Lineage 2	Livestock	MX	SLRRAPUS04-2
SAMEA5867066_L_MX_2003	5	Lineage 2	Livestock	MX	MIPUS03-27
SAMN09388826_H_UK_2016	5	Lineage 2	Human	UK	
SAMN02843813_E_MX_2005	5	Lineage 2	Plant	MX	
SAMN16248706_H_CA_2009	5	Lineage 2	Human	CA	
SAMN06030001_H_CA_UNK	5	Lineage 2	Human	CA	
SAMN20181544_H_CA_2017	5	Lineage 2	Human	CA	

SAMN08104946_H_US_2017	5	Lineage 2	Human	US	
SAMN05596256_UNK_US_UNK	5	Lineage 2	UNK	US	
SAMN06457912_UNK_US_UNK	5	Lineage 2	UNK	US	
SAMN05907722_H_US_2014	5	Lineage 2	Human	US	
SAMN05596124_UNK_US_UNK	5	Lineage 2	UNK	US	
SAMN20181504_H_CA_2016	5	Lineage 2	Human	CA	
SAMN18350908_H_CA_2018	5	Lineage 2	Human	CA	
SAMN18314377_H_CA_2018	5	Lineage 2	Human	CA	
SAMEA104280938_L_MX_2003	5	Lineage 2	Livestock	MX	SLPUS03-29
SAMEA104280918_H_MX_2003	5	Lineage 2	Human	MX	SLHS03-9
SAMEA104280084_H_MX_2003	5	Lineage 2	Human	MX	SLHS03-10
SAMN02368963_H_US_2007	5	Lineage 2	Human	US	
SAMEA7112700_P_MX_2003	5	Lineage 2	Poultry	MX	MIPLOS03-75
SAMEA104280107_H_MX_2003	5	Lineage 2	Human	MX	YUHS03-19
SAMN27058402_E_MX_2020	5	Lineage 2	Water/River	MX	
SAMN12364798_UNK_US_UNK	5	Lineage 2	UNK	US	
SAMN10715264_H_US_2010	5	Lineage 2	Human	US	
SAMN04394891_H_US_2010	5	Lineage 2	Human	US	
SAMN11309560_H_US_2017	5	Lineage 2	Human	US	
SAMN08054986_H_US_UNK	5	Lineage 2	Human	US	
SAMN10975460_H_US_2010	5	Lineage 2	Human	US	
SAMN12205280_UNK_US_UNK	5	Lineage 2	UNK	US	
SAMN09283748_UNK_US_UNK	5	Lineage 2	UNK	US	
SAMN08412589_H_US_2015	5	Lineage 2	Human	US	
SAMN07830924_UNK_US_UNK	5	Lineage 2	UNK	US	
SAMN11808362_UNK_US_UNK	5	Lineage 2	UNK	US	
SAMN07419599_UNK_US_UNK	5	Lineage 2	UNK	US	
SAMN11939925_UNK_US_UNK	5	Lineage 2	UNK	US	
SAMN11391575_UNK_US_UNK	5	Lineage 2	UNK	US	
SAMN07998287_UNK_US_UNK	5	Lineage 2	UNK	US	
SAMN02911984_H_US_2012	5	Lineage 2	Human	US	
SAMN09381875_UNK_US_UNK	5	Lineage 2	UNK	US	
SAMN27110635_E_MX_2021	5	Lineage 2	Water/River	MX	
SAMN06099712_H_US_2016	5	Lineage 2	Human	US	

SAMN07159154_H_US_2017	5	Lineage 2	Human	US	
SAMN04526241_H_US_2015	5	Lineage 2	Human	US	
SAMN04526240_H_US_2015	5	Lineage 2	Human	US	
SAMN04526239_H_US_2015	5	Lineage 2	Human	US	
SAMN16551987_L_US_2020	5	Lineage 2	Livestock	US	
SAMN23213223_L_US_2021	5	Lineage 2	Livestock	US	
SAMN14125598_L_US_2019	5	Lineage 2	Livestock	US	
SAMN05596705_UNK_US_UNK	5	Lineage 2	UNK	US	
SAMN20181555_H_CA_2017	5	Lineage 2	Human	CA	
SAMN13981981_E_MX_2019	5	Lineage 2	Water/River	MX	
SAMN09479056_UNK_US_UNK	5	Lineage 2	UNK	US	
SAMN08567745_H_US_2017	5	Lineage 2	Human	US	
SAMN10734131_H_US_2012	5	Lineage 4	Human	US	
SAMN04394819_H_US_2012	5	Lineage 4	Human	US	
SAMN14007870_E_MX_2019	5	Lineage 4	Water/River	MX	
SAMN14007859_E_MX_2019	5	Lineage 4	Water/River	MX	
SAMN27110705_E_MX_2021	5	Lineage 4	Water/River	MX	
SAMN27110657_E_MX_2021	5	Lineage 4	Water/River	MX	
SAMN07260218_UNK_US_UNK	5	Lineage 4	UNK	US	
SRR6879627_H_US_2018	5	Lineage 4	Human	US	
SAMN08773243_H_US_2018	5	Lineage 4	Human	US	
SAMN20427104_UNK_US_2021	5	Lineage 4	UNK	US	
SAMN10058523_H_UK_2018	5	Lineage 4	Human	UK	
SAMN07258306_H_US_2017	5	Lineage 4	Human	US	
SAMN14008526_E_MX_2019	5	Lineage 4	Water/River	MX	
SAMN14008532_E_MX_2019	5	Lineage 4	Water/River	MX	
SAMN14008529_E_MX_2019	5	Lineage 4	Water/River	MX	
SAMN14008536_E_MX_2019	5	Lineage 4	Water/River	MX	
SAMN14008521_E_MX_2019	5	Lineage 4	Water/River	MX	
SAMN14008522_E_MX_2019	5	Lineage 4	Water/River	MX	
SAMN14008530_E_MX_2019	5	Lineage 4	Water/River	MX	
SAMN14008510_E_MX_2019	5	Lineage 4	Water/River	MX	
SAMEA104280068_H_MX_2002	5	Lineage 2	Human	MX	MIHS02-19
SAMN10076456_L_MX_2004	5	Lineage 3	Livestock	MX	SL26

SAMEA6514417_L_MX_2004	5	Lineage 3	Livestock	MX	SLRARES04-8
SAMN12392219_UNK_US_UNK	5	Lineage 3	UNK	US	
SAMN07647615_UNK_US_UNK	5	Lineage 3	UNK	US	
SAMN16248710_H_CA_2010	5	Lineage 3	Human	CA	
SAMN08898437_UNK_US_UNK	5	Lineage 3	UNK	US	
SAMN09396828_H_UK_2017	5	Lineage 3	Human	UK	
SAMN08981725_UNK_US_UNK	5	Lineage 3	UNK	US	
SAMN06927756_UNK_US_UNK	5	Lineage 3	UNK	US	
SAMN09604470_UNK_US_UNK	5	Lineage 3	UNK	US	
SAMN05525302_UNK_US_UNK	5	Lineage 3	UNK	US	
SAMN10715260_H_US_2011	5	Lineage 3	Human	US	
SAMN04394917_H_US_2011	5	Lineage 3	Human	US	
SAMN04394774_H_US_2011	5	Lineage 3	Human	US	
SAMN06677648_H_US_2017	5	Lineage 3	Human	US	
SAMEA7114277_H_MX_2004	5	Lineage 3	Human	MX	YUHS04-50
SAMEA104279715_H_MX_2003	5	Lineage 3	Human	MX	YUHS03-72
SAMEA104280572_H_MX_2002	5	Lineage 3	Human	MX	SLHS02-12
SAMEA104280118_H_MX_2003	5	Lineage 3	Human	MX	YUHS03-34
SAMEA7113755_H_MX_2003	5	Lineage 3	Human	MX	YUHS03-31
SAMEA104280052_H_MX_2003	5	Lineage 3	Human	MX	YUHSO3-84
SAMEA104280480_H_MX_2005	5	Lineage 3	Human	MX	YUHS05-26
SAMN16248691_H_CA_2008	5	Lineage 3	Human	CA	
SAMN08163899_H_US_2014	5	Lineage 3	Human	US	
SAMN02368952_H_US_2007	5	Lineage 3	Human	US	
SAMEA104280473_H_MX_2003	5	Lineage 3	Human	MX	YUHS03-25
SAMEA6514427_L_MX_2003	5	Lineage 3	Livestock	MX	YUPUS03-18
SAMEA104280578_L_MX_2003	5	Lineage 3	Livestock	MX	YURES03-7
SAMEA104280105_H_MX_2004	5	Lineage 3	Human	MX	YUHS04-23
SAMEA104279739_H_MX_2004	5	Lineage 3	Human	MX	YUHS04-62
SAMEA5867114_H_MX_2002	5	Lineage 3	Human	MX	YUHS02-75
SAMEA104280941_H_MX_2003	5	Lineage 3	Human	MX	YUHS03-58A
SAMEA6514399_H_MX_2004	5	Lineage 3	Human	MX	YUHS04-39
SAMEA104279699_H_MX_2003	5	Lineage 3	Human	MX	YUHS03-26
SAMN17949861_H_CA_2008	5	Lineage 3	Human	CA	

SAMN03653812_H_MX_2005	5	Lineage 3	Human	MX	YUHS05-78
SAMN07625251_UNK_US_UNK	5	Lineage 3	UNK	US	
SAMN07141734_L_US_2017	5	Lineage 3	Livestock	US	
SAMEA104279921_H_UK_2004	5	Lineage 3	Human	UK	
SAMN09294231_UNK_US_UNK	5	Lineage 3	UNK	US	
SAMEA104280054_H_MX_2004	5	Lineage 3	Human	MX	YUHS04-15
SAMN10076457_H_MX_2007	5	Lineage 3	Human	MX	YUHS07-18
SAMN19006541_H_CA_2018	5	Lineage 3	Human	CA	
SAMEA5867052_H_MX_2004	5	Lineage 3	Human	MX	YUHS04-36
SAMEA104279675_H_MX_2004	5	Lineage 3	Human	MX	YUHS04-31
SAMEA6514405_H_MX_2005	5	Lineage 3	Human	MX	YUHS05-75
SAMN10715267_H_US_2010	5	Lineage 3	Human	US	
SAMN07688336_UNK_US_UNK	5	Lineage 3	UNK	US	
SAMN18618518_H_AU_2017	5	Lineage 3	Human	AU	
SAMN08784402_H_US_2018	5	Lineage 3	Human	US	

Supplementary_Table_2

Strain or plasmid	Genotype or description	Ref.
E. coli S17-1 λpir	Sm^R (λpir)	1
14028	WT, *Sm* ^{R}	Laboratory collection
14028 Δ*ompD*	Δ*ompD* ::*Cm* ^{R}	2
YUHS-05-78	WT	3
YUHS-05-78 Δ*ompD*	Δ*ompD*	This study
YUHS-05-78::*ompD*	*ompD* +	This study
YUHS-07-18	WT	4
YUHS-07-18 Δ*ompD*	Δ*ompD*	This study
YUHS-07-18::*ompD*	*ompD* +	This study
pRE118 plasmid	ori R6K, *Km* ^{R}	5

Primer	Sequence (5'-3')	Observations	Restriction sites
Fw1-ompD-vs	cgtaGAGCTCgggtcttattttacccccgtcatg	Deletion *ompD* / Complementation *ompD*	SacI
Rv1-ompD-vs	cgtcAAGCTTcactgccactaacttaagtttcat	Deletion *ompD*	HindIII
Fw2-ompD-vs	tgctAAGCTTgttggtctgaactaccagttctaa	Deletion *ompD*	HindIII
Rv2-ompD-vs	atTCTAGAtttatctggtcgcatagcgtg	Deletion *ompD* / Complementation *ompD*	XbaI

1. Simon, R., Priefer, U. & Pühler, A. A Broad Host Range Mobilization System for In Vivo Genetic Engineering: Transposon Mutagenesis in Gram Negative Bacteria. *Nat Biotechnol* **1**, 784–791 (1983).

2. Ipinza, F. *et al.* Participation of the Salmonella OmpD porin in the infection of RAW264.7 macrophages and BALB/c mice. *PLoS One* **9**, e111062 (2014).

3. Zaidi, M. B. *et al.* Integrated food chain surveillance system for Salmonella spp. in Mexico. *Emerging infectious diseases* **14**, 429–35 (2008).

4. Zaidi, M. B. *et al.* Burden and transmission of zoonotic foodborne disease in a rural community in Mexico. *Clin Infect Dis* **55**, 51–60 (2012).

5. Edwards, R. A., Keller, L. H. & Schifferli, D. M. Improved allelic exchange vectors and their use to analyze 987P fimbria gene expression. *Gene* **207**, 149–57 (1998).
