# Supplementary material for: Population structure and ongoing microevolution of the emerging multidrug-resistant Salmonella Typhimurium ST213
Source: NPJ Antimicrob Resist. 2024 Apr 8;2:10. doi: 10.1038/s44259-024-00027-6 (PMC11721120; doi:10.1038/s44259-024-00027-6)
Supplement: Supplementary file 3 — Reporting Summary [file 44259_2024_27_MOESM3_ESM.pdf]

nature portfolio

Corresponding author(s): Serrano-Fujarte Isela, Puente José Luis

Last updated by author(s): Nov 26, 2023

Reporting Summary

Nature Portfolio wishes to improve the reproducibility of the work that we publish. This form provides structure for consistency and transparency in reporting. For further information on Nature Portfolio policies, see our Editorial Policies and the Editorial Policy Checklist.

Statistics

For all statistical analyses, confirm that the following items are present in the figure legend, table legend, main text, or Methods section.

n/a	Confirmed	
☐	☒	The exact sample size (*n*) for each experimental group/condition, given as a discrete number and unit of measurement
☐	☒	A statement on whether measurements were taken from distinct samples or whether the same sample was measured repeatedly
☐	☒	The statistical test(s) used AND whether they are one- or two-sided<br>*Only common tests should be described solely by name; describe more complex techniques in the Methods section.*
☒	☐	A description of all covariates tested
☒	☐	A description of any assumptions or corrections, such as tests of normality and adjustment for multiple comparisons
☒	☐	A full description of the statistical parameters including central tendency (e.g. means) or other basic estimates (e.g. regression coefficient) AND variation (e.g. standard deviation) or associated estimates of uncertainty (e.g. confidence intervals)
☒	☐	For null hypothesis testing, the test statistic (e.g. *F*, *t*, *r*) with confidence intervals, effect sizes, degrees of freedom and *P* value noted<br>*Give P values as exact values whenever suitable.*
☒	☐	For Bayesian analysis, information on the choice of priors and Markov chain Monte Carlo settings
☒	☐	For hierarchical and complex designs, identification of the appropriate level for tests and full reporting of outcomes
☒	☐	Estimates of effect sizes (e.g. Cohen's *d*, Pearson's *r*), indicating how they were calculated

Our web collection on statistics for biologists contains articles on many of the points above.

Software and code

Policy information about availability of computer code

Data collection	Microsoft Excel 365 for Windows 10 version 2310.

Data analysis

PhaME pipeline (v1.0.2)
MEGA (v11)
panaroo (v1.3.4)
MAFFT (v7.520)
SNP-sites (v.2.5.1)
FastBAPs (v1.0.8)
ggtree (v3.4.0)
RStudio (v2022.07.2+576)
R base version 4.2.1
CSI phylogeny (v1.4)
TempEst (v1.5.3)
IQTREE (v2.0.3)
iToL (v6)
ggplot2 (v3.3.6)
Fiji (v2.35)
Tidyverse collection (v1.3.1)
Corrplot (v0.92)
ggpubr (v0.4.4)

For manuscripts utilizing custom algorithms or software that are central to the research but not yet described in published literature, software must be made available to editors and reviewers. We strongly encourage code deposition in a community repository (e.g. GitHub). See the Nature Portfolio guidelines for submitting code & software for further information.

Data

Policy information about availability of data

All manuscripts must include a data availability statement. This statement should provide the following information, where applicable:

- Accession codes, unique identifiers, or web links for publicly available datasets
- A description of any restrictions on data availability
- For clinical datasets or third party data, please ensure that the statement adheres to our policy

The data that support the findings are available as supplementary material. The files for the tree building, RDAR raw images, other raw data, and R scripts for the analysis and graphics are available upon request from the authors .

Research involving human participants, their data, or biological material

Policy information about studies with human participants or human data. See also policy information about sex, gender (identity/presentation), and sexual orientation and race, ethnicity and racism.

Reporting on sex and gender	Not applicable
Reporting on race, ethnicity, or other socially relevant groupings	Not applicable
Population characteristics	Not applicable
Recruitment	Not applicable
Ethics oversight	Not applicable

Note that full information on the approval of the study protocol must also be provided in the manuscript.

Field-specific reporting

Please select the one below that is the best fit for your research. If you are not sure, read the appropriate sections before making your selection.

☐ Life sciences ☐ Behavioural & social sciences ☒ Ecological, evolutionary & environmental sciences

For a reference copy of the document with all sections, see nature.com/documents/nr-reporting-summary-flat.pdf

Ecological, evolutionary & environmental sciences study design

All studies must disclose on these points even when the disclosure is negative.

Study description	The study outlines the population structure and genomic characterization of the emergent multidrug-resistant S. Typhimurium ST213, and it also incorporates the phenotypic characterization of ten representative isolates.
Research sample	The genomic data was recovered from the EnteroBase and included strains collected from 13 countries, Australia (2), Belgium (1),

Research sample	Canada (22), Denmark (5), France (2), India (3), Mexico (61), Netherlands (1), Portugal (2), Thailand (6), United Kingdom (71), USA (95) and Vietnam (4). In general, the 275 isolates include 161 isolates from humans, 37 form animals, 11 from food, 1 from plants, 16 from water/rivers and 49 from an unknown source.
Sampling strategy	Taking into account that the main aim of the study was to describe the population structure of the emerging ST213 strains, we considered all the genomic sequences publicly available at the time of the study. Therefore the sample size was sufficient at this time.
Data collection	The genomic data and metadata was recovered from the EnteroBase public database and the information was organized using excel.
Timing and spatial scale	The samples were isolated between 1957 and 2022 from 13 countries, Australia (2), Belgium (1), Canada (22), Denmark (5), France (2), India (3), Mexico (61), Netherlands (1), Portugal (2), Thailand (6), United Kingdom (71), USA (95) and Vietnam (4).<br>To provide a contextual framework for the study of the ST213 strains, we included all the isolates available in the sample in the study.
Data exclusions	Duplicated records in EnteroBase were excluded, and we were also unable to include non-publicly available sequences.
Reproducibility	Bioinformatic analyses were tested with at least two methods and proved to be reproducible, as indicated in the methods section, and the data are shown in supplementary figures.<br><br>The phenotypic characterizations were conducted at least three times independently and were reproducible.
Randomization	The primary aim of this study was to examine all the ST213 strains accessible in public records; therefore, there is no requirement to randomize the dataset.
Blinding	The metadata contained in the public records was utilized to describe and contextualize the work; therefore, in that aspect, no blinding was employed.

Did the study involve field work? ☐ Yes ☒ No

Reporting for specific materials, systems and methods

We require information from authors about some types of materials, experimental systems and methods used in many studies. Here, indicate whether each material, system or method listed is relevant to your study. If you are not sure if a list item applies to your research, read the appropriate section before selecting a response.

Materials & experimental systems

n/a	Involved in the study	
☒	☐	Antibodies
☐	☒	Eukaryotic cell lines
☒	☐	Palaeontology and archaeology
☒	☐	Animals and other organisms
☒	☐	Clinical data
☒	☐	Dual use research of concern
☒	☐	Plants

Methods

n/a	Involved in the study	
☒	☐	ChIP-seq
☒	☐	Flow cytometry
☒	☐	MRI-based neuroimaging

Eukaryotic cell lines

Policy information about cell lines and Sex and Gender in Research

Cell line source(s)	C2BBe1 (CRL-2102™) from ATCC®<br>HT-29 (HTB-38™) from ATCC®<br>THP-1 (TIB-2™) donated and previously obtainted from ATCC®.<br>U-937 (CRL-1593.2™) from ATCC®<br>Mouse embryonic fibroblasts derived from male mouse, donated.
Authentication	None
Mycoplasma contamination	None
Commonly misidentified lines<br>(See ICLAC register)	None

Plants

Seed stocks	Not applicable
Novel plant genotypes	Not applicable
Authentication	Not applicable
